# TGF*β* pathway limits dedifferentiation following WNT and MAPK pathway activation to suppress intestinal tumourigenesis

**DOI:** 10.1038/cdd.2017.92

**Published:** 2017-06-16

**Authors:** Patrizia Cammareri, David F Vincent, Michael C Hodder, Rachel A Ridgway, Claudio Murgia, Max Nobis, Andrew D Campbell, Julia Varga, David J Huels, Chithra Subramani, Katie L H Prescott, Colin Nixon, Ann Hedley, Simon T Barry, Florian R Greten, Gareth J Inman, Owen J Sansom

**Affiliations:** 1 Wnt Signaling and Colorectal Cancer Group, Cancer Research UK Beatson Institute, Garscube Estate, Glasgow G61 1BD, UK; 2 Institute for Tumor Biology and Experimental Therapy, Georg-Speyer-Haus, Frankfurt 60596 Germany; 3 Oncology IMED, AstraZeneca, Alderley Park SK10 4TG, Cambridge, UK; 4 German Cancer Consortium (DKTK) and German Cancer Research Center (DKFZ), Heidelberg 69120, Germany; 5 Division of Cancer Research, School of Medicine, University of Dundee, Dundee DD1 9SY, UK; 6 Institute of Cancer Sciences, University of Glasgow, Garscube Estate, Glasgow G61 1QH, UK

## Abstract

Recent studies have suggested increased plasticity of differentiated cells within the intestine to act both as intestinal stem cells (ISCs) and tumour-initiating cells. However, little is known of the processes that regulate this plasticity. Our previous work has shown that activating mutations of *Kras* or the NF-*κ*B pathway can drive dedifferentiation of intestinal cells lacking *Apc*. To investigate this process further, we profiled both cells undergoing dedifferentiation *in vitro* and tumours generated from these cells *in vivo* by gene expression analysis. Remarkably, no clear differences were observed in the tumours; however, during dedifferentiation *in vitro* we found a marked upregulation of TGF*β* signalling, a pathway commonly mutated in colorectal cancer (CRC). Genetic inactivation of TGF*β* type 1 receptor (*Tgfbr1/Alk5*) enhanced the ability of *Kras*^*G12D/+*^ mutation to drive dedifferentiation and markedly accelerated tumourigenesis. Mechanistically this is associated with a marked activation of MAPK signalling. Tumourigenesis from differentiated compartments is potently inhibited by MEK inhibition. Taken together, we show that tumours arising in differentiated compartments will be exposed to different suppressive signals, for example, TGF*β* and blockade of these makes tumourigenesis more efficient from this compartment.

Since the description of ‘top-down’ adenoma formation of colorectal tumours there has been discussion over the cell of origin of colorectal cancer (CRC). Here, human dysplastic crypts, carrying mutations of the *APC* tumour suppressor gene, were detectable exclusively at the top part of colonic crypts in patients with adenomatous polyps.^1^ Importantly, cells at the base of the crypts, on which the polyps were located, were morphologically normal and did not carry an *APC* mutation. However, histopathological analyses have also suggested a stem cell route through to the identification of monocryptal adenomas in patients who develop familial adenomatous polyposis due to a germline mutation in *APC*, supporting the ‘bottom-up’ model of intestinal tumourigenesis.^2^

To elucidate the cell of origin of human CRC, many studies have used ‘proof-of-concept’ approaches using genetically engineered mice to target different cell types and assess if they initiate tumourigenesis. This extensive work has also suggested two routes of intestinal cancer. In support of the ‘bottom-up’ model, one efficient route is where intestinal stem cells (ISCs) are transformed via *Apc* loss or stable expression of *β*-catenin and very rapidly form adenomas.^3, 4, 5^ At the same time, experimental evidence also supports the ‘top-down’ model of intestinal tumourigenesis, where non-ISCs are transformed and serve as cells of origin. However, non-ISCs alongside *Apc* loss require additional events to initiate tumourigenesis.^6, 7, 8^ Activation of oncogenic *Kras*^*G12D/+*^ or NF-*κ*B activation concomitantly with *Apc* loss can drive a dedifferentiation programme within the villus epithelium.^6^ It has also been shown that the long-lived differentiated DCLK1^+ve^ Tuft cells can act as a cell of origin for intestinal cancer^8^ if *Apc* loss was combined with inflammation. Moreover, elegant work by Davis *et al.*^9^ showed that the stromal microenvironment has a critical role in suppressing dedifferentiation. Disruption of the Gremlin-1 morphogen gradient within the intestinal epithelium led to the formation of ‘crypts in villus’, indicating that *Apc*-deficient enterocytes indeed dedifferentiated.

Recent sequencing studies have confirmed that in CRC alongside *APC* mutation there are common mutations in *KRAS*, *TP53* and *PI3KCA* genes. Additionally, the TGF*β* family members are also very commonly mutated or deregulated.^10^ Deletion, downregulation or inactivating mutations can occur in *TGFBR1*, *TGFBR2* and the SMAD effectors (*SMAD2–4*).^11, 12, 13^ Frameshift mutations in *TGFBR2* have been predominantly identified in CRC with mismatch repair (MMR) defects.^14^ In tumours with functional MMR, TGF*β* signalling is commonly abolished by loss of SMAD4.^11, 15^

In addition to this clear tumour-suppressive role, TGF*β* signalling also has tumour-promoting effects in the late stages of CRC. Enhanced levels of TGF*β* ligand expression has been associated with advanced tumours,^16^ and the activation of an epithelial-to-mesenchymal transition (EMT) programme.^17, 18^ High levels of TGF*β* target genes, in the stromal cells of CRC patients, are associated with poor prognosis,^19^ and in the recent publication of the consensus molecular subtypes of human CRC, high levels of TGF*β* signalling define a mesenchymal subtype with the poorest prognosis.^20^

Since TGF*β* signalling is commonly deregulated in CRC, we functionally explored the importance of this pathway and found that the likely driving force, behind these mutations, is the inhibition of its prodifferentiation tumour-suppressive effect. Mechanistically further deregulation of MAPK signalling is important for this phenotype.

## Results

### Transcriptome profiling of tumours derived from crypts *versus* villi failed to reveal major differences

**Figure 1:**
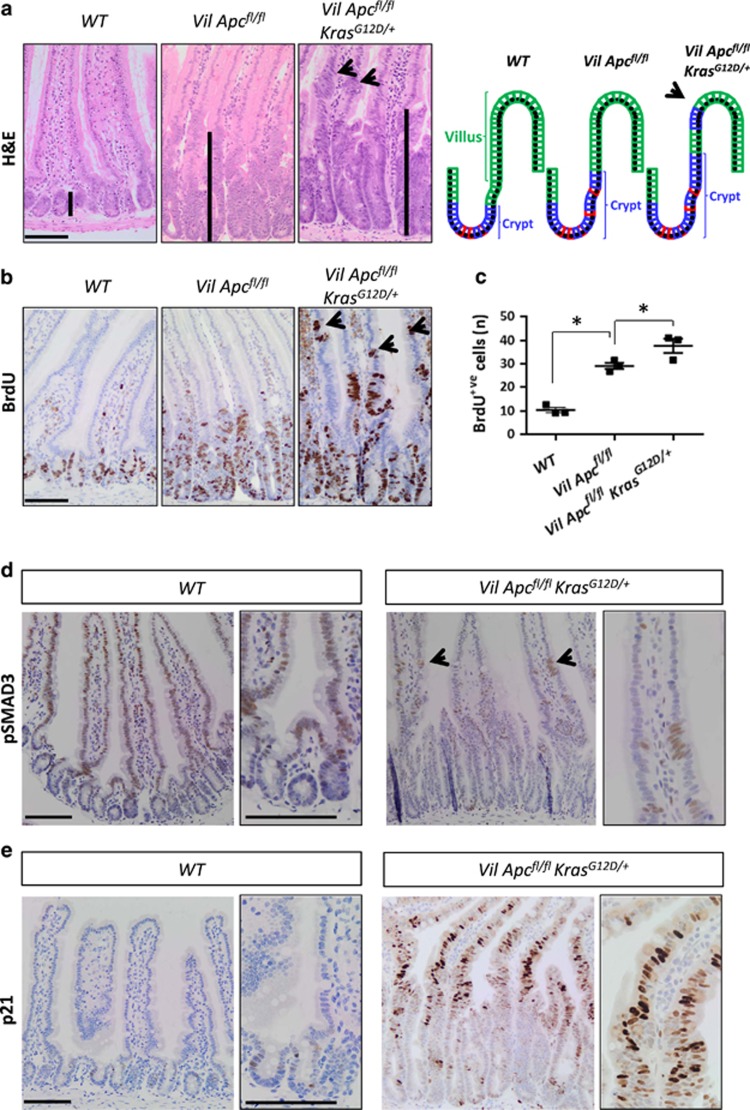
*Kras*^*G12D/+*^ enhances both the crypt-progenitor phenotype and transforming growth factor-*β* (TGF*β*) pathway activation following *Apc* loss. (**a**) Haematoxylin and eosin (H&E) staining and a schematic model of the crypt-progenitor phenotype of wild-type (*WT*), *VilCre*^*ER*^*Apc*^*fl/fl*^ (*Vil* *Apc*^*fl/fl*^) and *VilCre*^*ER*^*Apc*^*fl/fl*^*Kras*^*G12D/+*^ (*Vil* *Apc*^*fl/fl*^*Kras*^*G12D/+*^) small intestine. Note the presence of dedifferentiated cells (black arrow) in the villus of the *Vil* *Apc*^*fl/fl*^*Kras*^*G12D/+*^genotype. Black bars highlight the differences in crypt length. (**b**) Bromodeoxyuridine (BrdU) staining of *WT*, *Vil* *Apc*^*fl/f*^ and *Vil* *Apc*^*fl/fl*^*Kras*^*G12D/+*^ small intestine. Note the presence of proliferating cells (black arrow) in the villi of the *Vil* *Apc*^*fl/fl*^*Kras*^*G12D/+*^genotype. (**c**) Quantification of total BrdU+ cells demonstrating increased proliferation in the *Vil Apc*^*fl/fl*^*Kras*^*G12D/+*^ genotype. Error bars represent mean± S.E.M., *P=0.04 Mann–Whitney test, one-tailed, *n*=3 biological replicates. (**d**) pSMAD3 and (**e**) p21 IHC analysis of intestine from *WT* and *Vil* *Apc*^*fl/fl*^*Kras*^*G12D/+*^ mice. Black arrows indicate positive cells in the villi. High magnification insets highlight the presence of positive cells. Scale bars, 100 *μ*m

Acute deletion of *Apc* in the intestinal epithelium, using *VillinCre*^*ER*^ (referred to from now on as *VilCre*^*ER*^*Apc*^*fl/fl*^), causes a crypt-progenitor phenotype characterized by enlarged intestinal crypts when compared with the wild-type intestine (Figure 1a). An additional oncogenic mutation in *Kras* (referred to from now on as *VilCre*^*ER*^*Apc*^*fl/fl*^*Kras*^*G12D/+*^) results in a marked increase in crypt size (Figure 1a) with the presence of proliferative cells in the villus, which are not detected in wild-type or *VilCre*^*ER*^*Apc*^*fl/fl*^ mice (Figures 1b and c), showing that the additional mutation of *Kras* can drive proliferation in differentiated enterocytes.

In our previous studies, we have demonstrated that villi purified from *VilCre*^*ER*^*Apc*^*fl/fl*^*Kras*^*G12D/+*^ mice grow *in vitro* as spheroids with tumourigenic capacity (unlike villi purified from either wild-type or *VilCre*^*ER*^*Apc*^*fl/fl*^ mice).^6^ To investigate this process further, we analysed the transcriptomes of tumours derived through subcutaneous injection of either spheroids derived from crypts (CDS) or Villi (VDS) purified from *VilCre*^*ER*^*Apc*^*fl/fl*^*Kras*^*G12D/+*^ mice. Surprisingly, our microarray analysis revealed no consistent differences between tumours derived from injection of CDS or VDS (Supplementary Data 1), indicating that in these models the cell of origin did not modify the transcriptional profile of the tumours. This also suggested that dedifferentiation, provoked by WNT activation and *Kras*^*G12D/+*^ mutation, was complete and that, after transplantation, the cell of origin does not affect subsequent gene expression. To investigate this in an autochthonous environment, we then compared gene expression when WNT pathway activation (using a *β*-catenin mutant that cannot be targeted for degradation – referred to as *Ctnnb*^*loxEx3/+*^), coupled with an oncogenic *Kras*^*G12D/+*^ mutation, was targeted to just differentiated cells, using *Xbp1sCre*^*ER*^, or to all cells, using *VilCre*^*ER*^. Remarkably, expression profiles from epithelial extractions (performed when intestines showed a similar crypt-progenitor phenotype, 7 days for *VilCre*^*ER*^ and 21 days for *Xbp1sCre*^*ER*^) did not reveal any significant differences, again suggesting that once cells had undergone dedifferentiation the gene expression pattern of the initiating cells is lost (Supplementary Data 2a and b).

### TGF*β* signalling pathway is upregulated following WNT and oncogenic KRAS activation

We reasoned that to have a better understanding of the pathways responsible for dedifferentiation, we would need to investigate transcriptional changes as the process was occurring. For this reason, we profiled VDS *versus* CDS, purified from *VilCre*^*ER*^*Apc*^*fl/fl*^*Kras*^*G12D/+*^ mice, while they were still in culture. This approach proved more successful, and we were able to observe significant deregulation of a number of different growth signalling pathways (fold-change cutoffs⩾1.5, *P*⩽0.05; Supplementary Figure 1a and Supplementary Data 3). It was particularly notable that numerous members of the TGF*β* family and superfamily were upregulated in the VDS compared with CDS (fold-change cutoffs⩾1.5; Supplementary Table 1 and Supplementary Data 3). To assess whether upregulation of TGF*β* signalling was also occurring *in vivo*, we examined the activity of TGF*β* signalling immediately after combined acute loss of *Apc* and *Kras*^*G12D/+*^ mutation. We detected phosphorylated SMAD3 (pSMAD3) positivity mainly in the villus aberrant foci when compared with the adjacent normal epithelium in *VilCre*^*ER*^*Apc*^*fl/fl*^*Kras*^*G12D/+*^ mice (Figure 1d and Supplementary Figure 1b). Furthermore, p21 (a mediator of TGF*β* signalling) was highly expressed in the *VilCre*^*ER*^*Apc*^*fl/fl*^*Kras*^*G12D/+*^ villi when compared with wild-type villi (Figure 1e). We also observed increased levels of *Smad7* (a canonical TGF*β* target gene) in *VilCre*^*ER*^*Apc*^*fl/fl*^*Kras*^*G12D/+*^ mice along the crypt–villus axis compared with wild-type intestine (Supplementary Figure 1c).

We then assessed the activation state of the TGF*β* signalling pathway in intestinal tumours developed in *VilCre*^*ER*^*Apc*^*fl/+*^*Kras*^*G12D/+*^ mice. These mice develop tumours in both the small and large intestine upon loss of the remaining wild-type *Apc* allele (Supplementary Figure 2a). Importantly, tumours from these mice resemble both the top-down (Supplementary Figures 2b and d) and bottom-up morphologies (Supplementary Figures 2c and d). Importantly, we detected high levels of pSMAD3 and p21 in the top-down lesions, indicating an activation of TGF*β* signalling (Supplementary Figures 2e and f). Tumours generated according to the bottom-up model show a morphogenic pSMAD3 gradient along the crypt–villus axis (Supplementary Figure 2g).

Thus, these results suggest that WNT and KRAS signalling can promote TGF*β* signalling, particularly in cells that are dedifferentiating at crypt–villus junctions.

### TGF*β* signalling in wild-type intestine is higher as cells differentiate but is not essential for homeostasis

**Figure 2:**
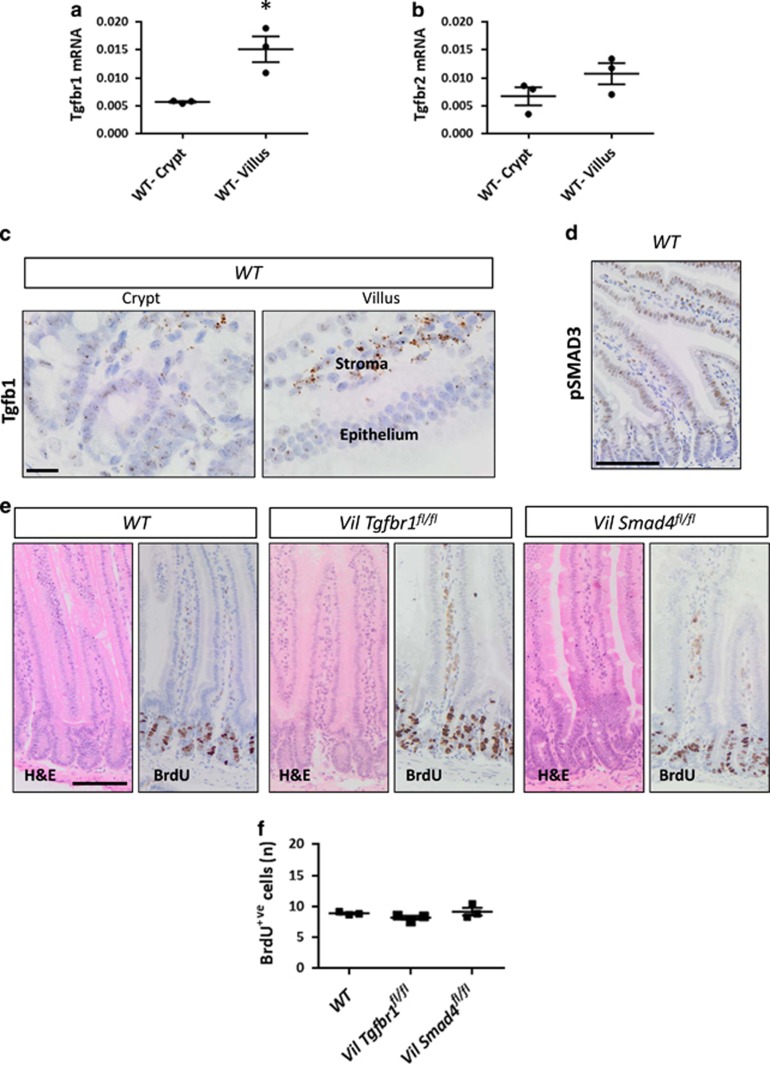
Loss of transforming growth factor-*β* (TGF*β*) signalling does not affect the normal homeostatic proliferation. (**a**) Quantitative real-time-PCR (qRT-PCR) analysis of *Tgfbr1* and (**b**) *Tgfbr2* expression in wild-type freshly purified crypts (WT-Crypt) *versus* villi (WT-Villus). Data are shown as ratios to the internal *Gapdh* control with error bars representing mean±S.E.M., *P=0.04 by Mann–Whitney test, one-tailed, *n*=3 biological replicates. (**c**) RNAscope analysis of *Tgfb1* in *WT* crypt and villus. Scale bars, 20 *μ*m. (**d**) IHC analysis of pSMAD3 on *WT* small intestine. (**e**) Haematoxylin and eosin (H&E) and bromodeoxyuridine (BrdU) staining of *WT*, *VilCre*^*ER*^*Tgfbr1*^*fl/fl*^ (*Vil* *Tgfbr1*^*fl/fl*^) and *VilCre*^*ER*^*Smad4*^*fl/fl*^ (*Vil Smad4*^*fl/fl*^) small intestine 4 days post induction with tamoxifen. Scale bars, 100 *μ*m. (**f**) Quantification of total BrdU+ cells in *WT*, *Vil* *Tgfbr1*^*fl/fl*^ and *Vil* *Smad4*^*fl/fl*^ small intestine. Error bars represent mean±S.E.M., *n*=3 biological replicates

The TGF*β* pathway has been previously reported to form a gradient along the vertical axis of the intestinal epithelium with the highest activity in differentiated cells and lowest activity in the crypt.^21^ However, studies that have genetically ablated TGF*β* signalling either via receptor or SMAD4 deletion, have not reported marked effects on homeostasis.^22, 23^ For our studies if tumour cells arise further up the crypt–villus axis, they may be exposed to a high level of TGF*β* signalling that might prevent dedifferentiation and limit proliferation. We first examined the expression of TGF*β* signalling components in wild-type intestine. We compared *Tgfbr1* and *Tgfbr2* mRNA expression in freshly purified crypts with villi (which include intravillus stromal cells). mRNA of both receptors was detectable in both compartments with higher levels in the villus (Figures 2a and b). The *Tgfb1* ligand expression was mainly detected in the intravillus region (Figure 2c), suggesting that the differentiated compartment might be exposed to more ligand and therefore activate TGF*β* signalling to a higher extent. We then stained wild-type intestines for pSMAD3. Nuclear pSMAD3 was mainly detected in the late transit-amplifying and differentiated cells (Figure 2d).

In line with previous studies, deletion of either *Tgfbr1* (*VilCre*^*ER*^*Tgfbr1*^*fl/fl*^) or the key downstream transcription factor SMAD4 (*VilCre*^*ER*^*Smad4*^*fl/fl*^) had no impact on intestinal proliferation. Normal levels of proliferation were observed 4 days after conditional deletion when compared with wild-type intestine (Figures 2e and f). Taken together, these data indicate that canonical TGF*β* signalling is higher in the differentiated compartment of the intestine and could have a role in suppressing dedifferentiation even in the absence of a homeostatic role.

### Loss of TGF*β* signalling is not sufficient to drive dedifferentiation if only *Apc* is deleted

**Figure 3:**
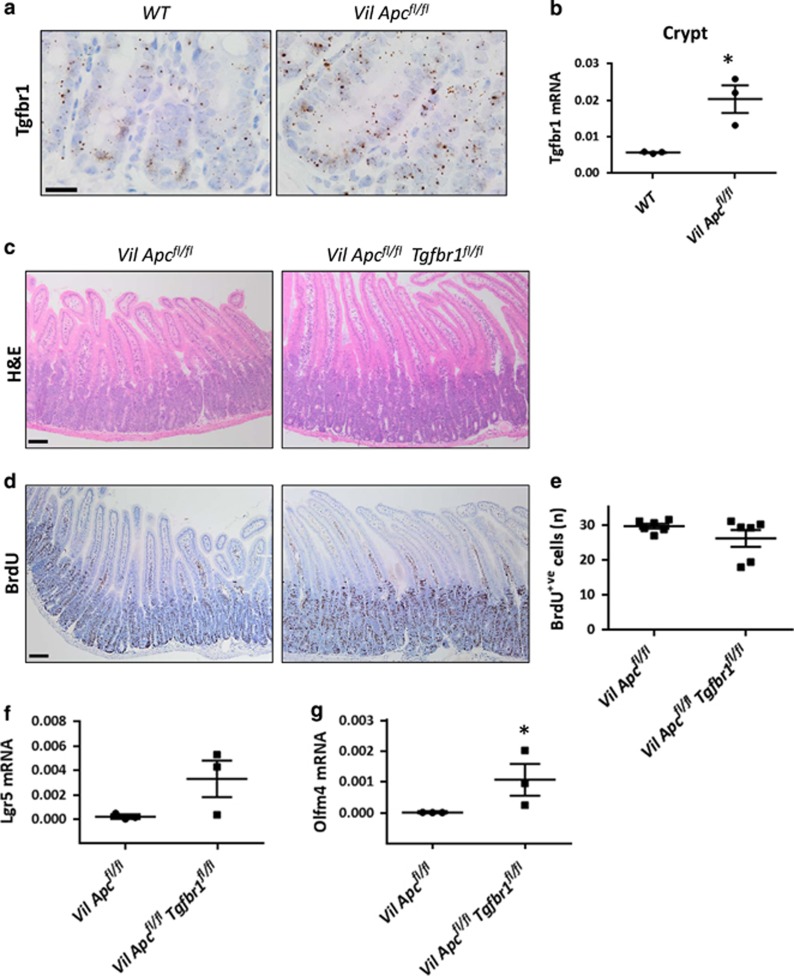
*Tgfbr1* is upregulated following *Apc* loss. (**a**) RNAscope of *Tgfbr1* on wild-type (*WT*) and *VilCre*^*ER*^*Apc*^*fl/fl*^ (*Vil* *Apc*^*fl/fl*^) crypts. Note the increased positivity following *Apc* loss. Scale bars, 20 *μ*m. (**b**) Quantitative real-time-PCR (qRT-PCR) analysis of *Tgfbr1* on *WT* and *Vil Apc*^*fl/fl*^ crypts. Data are shown as ratios to the internal *Gapdh* control with error bars representing mean±S.E.M., *P=0.04 by Mann–Whitney test, one-tailed, *n*=3 biological replicates. (**c**) Haematoxylin and eosin (H&E) and (**d**) bromodeoxyuridine (BrdU) staining on *Vil* *Apc*^*fl/fl*^ and *VilCre*^*ER*^*Apc*^*fl/fl*^*Tgfbr1*^*fl/fl*^ (*Vil* *Apc*^*fl/fl*^*Tgfbr1*^*fl/fl*^) intestine 4 days post induction with tamoxifen. Scale bars, 100 *μ*m. (**e**) Quantification of total BrdU+ cells of *Vil* *Apc*^*fl/fl*^ and *Vil* *Apc*^*fl/fl*^ *Tgfbr1*^*fl/fl*^ intestine. Error bars represent mean±S.E.M., *n*=6 biological replicates. (**f**) qRT-PCR analysis of the stem cell markers *Lgr5* and (**g**) *Olfm4* on CDS generated from *Vil* *Apc*^*fl/fl*^ and *Vil* *Apc*^*fl/fl*^*Tgfbr1*^*fl/fl*^ small intestine. Data are shown as ratios to the internal *Gapdh* control with error bars representing mean±S.E.M., *P=0.04 by Mann–Whitney test, one-tailed, *n*=3 biological replicates

Analysis of our previous microarray,^24^ comparing the transcriptomes of wild-type intestine to intestine where *Apc* has been acutely deleted, showed that *Tgfbr1* is upregulated following acute *Apc* loss. To confirm this, we analysed the expression of TGF*β* signalling components in *VilCre*^*ER*^*Apc*^*fl/fl*^ mice 4 days post induction and compared this with wild-type mice. We observed a marked increase in *Tgfbr1* mRNA levels in the crypts but not the villi (Figures 3a and b and Supplementary Figure 3a).

Similar to the wild-type intestine, stromal cells were the main source of TGF*β*1 (Supplementary Figure 3b). After *Apc* deletion, *Smad7* mRNA was detectable in both crypts and villi (Supplementary Figure 3c) and p21 was strongly induced at the crypt–villus transition zone (Supplementary Figure 3d).

To test the functional importance of TGF*β* signalling upregulation following *Apc* loss, we generated *VilCre*^*ER*^*Apc*^*fl/fl*^*Tgfbr1*^*fl/fl*^ mice and analysed the crypt-progenitor phenotype 4 days post induction. Surprisingly, crypt size and the number of proliferative cells were not increased by *Tgfbr1* loss (Figures 3c–e), suggesting that in the absence of *Kras* mutation, *Tgfbr1* deficiency was insufficient to drive dedifferentiation. Accordingly, villi purified from *VilCre*^*ER*^*Apc*^*fl/fl*^*Tgfbr1*^*fl/fl*^ mice were unable to form spheroids *in vitro* (Supplementary Figure 3e). Interestingly, *Tgfbr1* loss increased the mRNA expression levels of the stem cells markers *Lgr5* and *Olfm4* in CDS (Figures 3f and g). To functionally test this, we then generated single-cell clones and observed that *Tgfbr1* loss increased the number of cells with clonogenic potential. (Supplementary Figure 3f). These data are consistent with work by Batlle and co-workers^25^ who suggested that loss of TGF*β* signalling increases stem cell properties and that TGFBR1/ALK5 inhibitors are required for human colonic organoid cultures.

We then aged *VilCre*^*ER*^*Apc*^*fl/+*^*Tgfbr1*^*fl/fl*^ mice until they developed clinical signs. We observed a significant decrease in tumour latency compared with *VilCre*^*ER*^*Apc*^*fl/+*^ mice (Supplementary Figure 3g). However, in contrast to *VilCre*^*ER*^*Apc*^*fl/+*^*Kras*^*G12D/+*^ mice, there was no presence of top-down lesions. Consistent with previous studies,^22, 26^ the major consequence of *Tgfbr1* loss on tumourigenesis was the development of invasive adenocarcinomas with E-cadherin^+ve^ cells at the invasive front (Supplementary Figures 3h and i). Overall, these data show that while loss of TGF*β* can drive progression of adenomas to adenocarcinoma, it is not sufficient to drive dedifferentiation in the absence of the *Kras*^*G12D*^ mutation.

### Loss of TGF*β* signalling increases the crypt-progenitor phenotype and accelerates tumourigenesis when both *Apc* and *Kras* are mutated

**Figure 4:**
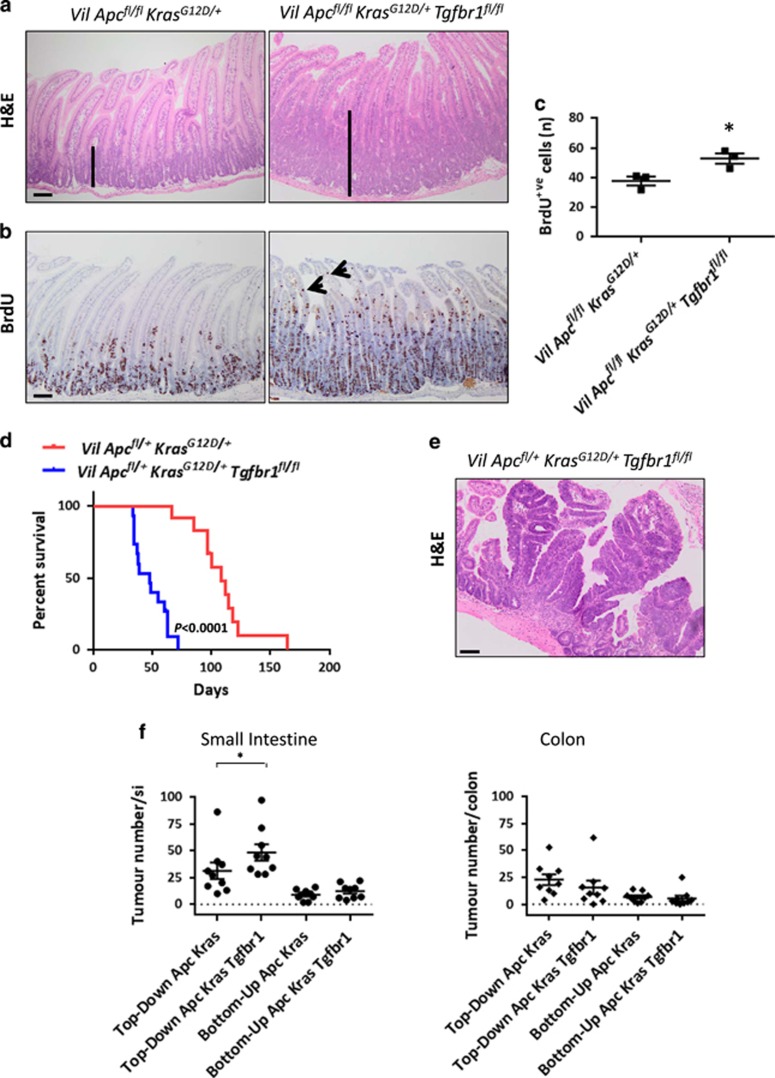
Loss of *Tgfbr1* cooperates with WNT and KRAS activation to accelerate intestinal tumourigenesis. (**a**) Haematoxylin and eosin (H&E) and (**b**) bromodeoxyuridine (BrdU) IHC on *VilCre*^*ER*^*Apc*^*fl/fl*^*Kras*^*G12D/+*^ (*Vil* *Apc*^*fl/fl*^*Kras*^*G12D/+*^) and *VilCre*^*ER*^*Apc*^*fl/fl*^*Kras*^*G12D/+*^*Tgfbr1*^*fl/fl*^ (*Vil* *Apc*^*fl/fl*^*Kras*^*G12D/+*^*Tgfbr1*^*fl/fl*^) small intestine 3 days post induction with tamoxifen. Black bars highlight the difference in the crypt length. Black arrows indicate the presence of proliferating cells along the villus in the *VilCre*^*ER*^*Apc*^*fl/fl*^*Kras*^*G12D/+*^*Tgfbr1*^*fl/fl*^ genotype. Scale bars, 100 *μ*m. (**c**) Quantification of the total BrdU+ cells per half crypt of the *Vil* *Apc*^*fl/fl*^*Kras*^*G12D/+*^ and *Vil* *Apc*^*fl/fl*^*Kras*^*G12D/+*^*Tgfbr1*^*fl/fl*^ small intestine. Error bars represent mean±S.E.M., *P=0.04 by Mann–Whitney test, one-tailed, *n*=3 biological replicates. (**d**) Kaplan–Meier survival curve of *VilCre*^*ER*^*Apc*^*fl/+*^*Kras*^*G12D/+*^ (*n*=12) and *VilCre*^*ER*^*Apc*^*fl/+*^*Kras*^*G12D/+*^*Tgfbr1*^*fl/fl*^ (*n*=15) mice, ****P<0.0001 by log-rank (Mantel–Cox) test. (**e**) H&E of top-down tumours in *VilCre*^*ER*^*Apc*^*fl/+*^*Kras*^*G12D/+*^*Tgfbr1*^*fl/fl*^ (*Vil* *Apc*^*fl/+*^*Kras*^*G12D/+*^ *Tgfbr1*^*fl/fl*^) mice. Scale bars, 100 *μ*m. (**f**) Quantification of total tumour number per small intestine and colon developed from crypt (bottom-up) or villus (top-down) in *VilCre*^*ER*^*Apc*^*fl/+*^*Kras*^*G12D/+*^ (Apc Kras) and *VilCre*^*ER*^*Apc*^*fl/+*^*Kras*^*G12D/+*^*Tgfbr1*^*fl/fl*^ (Apc Kras Tgfbr1) mice. Error bars represent mean±S.E.M., *P=0.023 by Mann–Whitney test, one-tailed

Given that *Kras* mutation appears to be critical for dedifferentiation and that we observed increased TGF*β* signalling in VDS compared with CDS, we generated the *VilCre*^*ER*^*Apc*^*fl/fl*^*Kras*^*G12D/+*^*Tgfbr1*^*fl/fl*^ mouse model to assess if this modified the acute crypt-progenitor phenotype of *VilCre*^*ER*^*Apc*^*fl/fl*^*Kras*^*G12D/+*^ mice. *Tgfbr1* loss now significantly increased the crypt-progenitor phenotype of *VilCre*^*ER*^*Apc*^*fl/fl*^*Kras*^*G12D/+*^ mice (Figure 4a). Importantly, proliferative cells were observed even further into the top part of the villus, suggesting enhanced proliferation (Figures 4b and c). Loss of TGF*β* signalling was again associated with an increased expression of *Lgr5* and *Olfm4* in CDS (Supplementary Figures 4a and b) and with an increased clonogenic potential (Supplementary Figure 4c). We also observed increased *Lgr5* and *Olfm4* expression levels in the crypts of *VilCre*^*ER*^*Apc*^*fl/fl*^*Kras*^*G12D/+*^*Tgfbr1*^*fl/fl*^ mice when compared with *VilCre*^*ER*^*Apc*^*fl/fl*^*Kras*^*G12D/+*^ mice. Whereas *Lgr5* and *Olfm4* levels in the villi were negligible at an early time point (Supplementary Figures 4d and e). To assess whether the increased crypt-progenitor phenotype observed in *VilCre*^*ER*^*Apc*^*fl/fl*^*Kras*^*G12D/+*^*Tgfbr1*^*fl/fl*^ mice translated into enhanced tumourigenesis, we generated cohorts of *VilCre*^*ER*^*Apc*^*fl/+*^*Kras*^*G12D/+*^ and *VilCre*^*ER*^*Apc*^*fl/+*^*Kras*^*G12D/+*^*Tgfbr1*^*fl/fl*^ mice and aged them until they showed clinical signs. *Tgfbr1* deletion accelerated intestinal tumourigenesis (Figure 4d). *VilCre*^*ER*^*Apc*^*fl/+*^*Kras*^*G12D/+*^*Tgfbr1*^*fl/fl*^ mice developed tumours both in the small intestine and colon. Importantly, many lesions were now observed with histological characteristics reminiscent of top-down lesions, suggesting that they had arisen from differentiated compartments (Figures 4e and f), and 50% of mice developed *in situ* invasive tumours (Supplementary Figure 4f). Overall, these data suggest that TGF*β* signalling acts as a potent tumour suppressor in the presence of WNT and RAS pathway activation, limiting proliferation, stem cell marker expression and potentially plasticity/dedifferentiation.

### Loss of TGF*β* signalling promotes dedifferentiation

**Figure 5:**
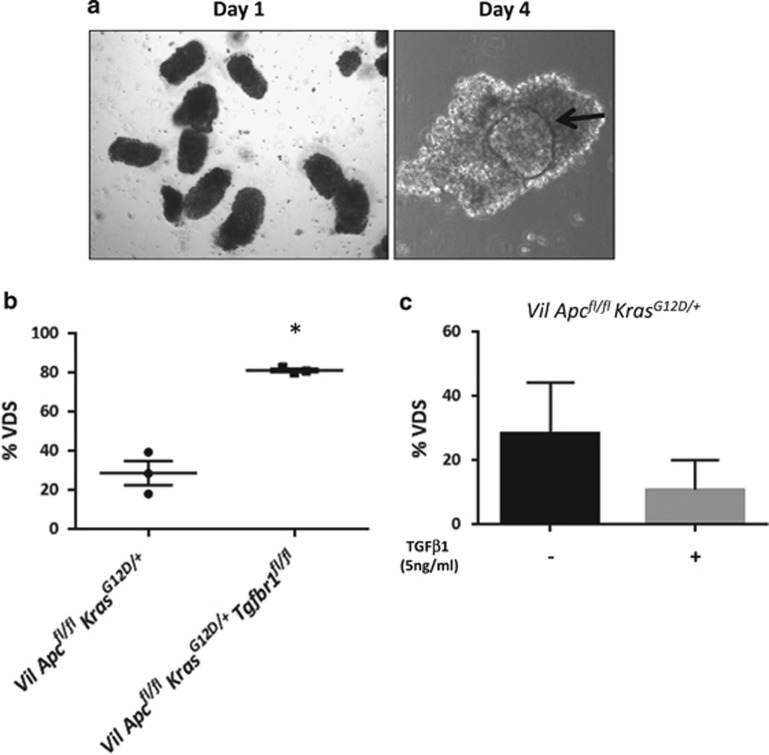
Transforming growth factor-*β* (TGF*β*) signalling attenuates the capacity of villi to dedifferentiate *in vitro.* (**a**) Morphology of villi freshly purified from *VilCre*^*ER*^*Apc*^*fl/fl*^*Kras*^*G12D/+*^ mice at day 1 (left panel) and day 4 (right panel) in culture. Black arrow indicates a spheroid inside the villus at day 4. (**b**) Quantification of VDS generated from *Vil* *Apc*^*fl/fl*^*Kras*^*G12D/+*^ and *VilCre*^*ER*^*Apc*^*fl/fl*^*Kras*^*G12D/+*^*Tgfbr1*^*fl/fl*^ (*Vil Apc*^*fl/fl*^*Kras*^*G12D/+*^*Tgfbr1*^*fl/fl*^) intestine. Error bars represent mean±S.E.M., *P=0.04 by Mann–Whitney test, one-tailed, *n*=3 biological replicates. (**c**) Quantification of VDS derived from *VilCre*^*ER*^*Apc*^*fl/fl*^*Kras*^*G12D/+*^ villi treated with TGF*β*1 (5 ng/ml) or Vehicle for 72 h. Plot represents mean with standard deviation (s.d.), *n*=3 biological replicates

To study the role of TGF*β* on dedifferentiation in more detail, we took advantage of the villus culture system. We purified villi from *VilCre*^*ER*^*Apc*^*fl/fl*^*Kras*^*G12D/+*^ and *VilCre*^*ER*^*Apc*^*fl/fl*^*Kras*^*G12D/+*^*Tgfbr1*^*fl/fl*^ mice and monitored the formation of organoids *in vitro*. *Tgfbr1* loss increased the efficacy of spheroid formation from villi (Figures 5a and b), confirming the role of TGF*β* in restraining dedifferentiation. *VilCre*^*ER*^*Apc*^*fl/fl*^*Kras*^*G12D/+*^*Tgfbr1*^*fl/fl*^ VDS can be propagated long term in culture and have tumourigenic potential. Allograft tumours showed strong expression of the stem cell markers *Lgr5* and CD44v6 (Supplementary Figures 5a–c).

To test whether increasing TGF*β* would further suppress dedifferentiation, we treated *VilCre*^*ER*^*Apc*^*fl/fl*^*Kras*^*G12D/+*^ villi with TGF*β*1 and found that exogenous TGF*β*1 reduced the capacity to form spheroids *in vitro* (Figure 5c). Taken together, these data confirmed that loss of TGF*β* signalling increases dedifferentiation.

### Loss of TGF*β* pathway increases pERK levels and induces sensitivity to a MEK1/2 inhibition

**Figure 6:**
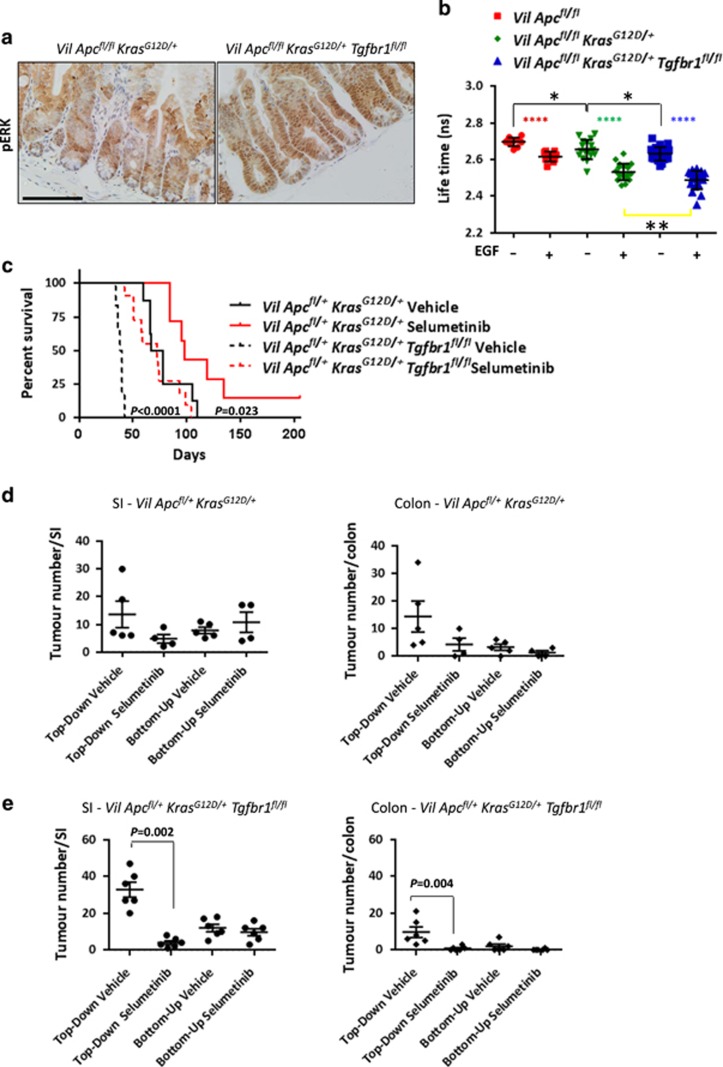
Loss of transforming growth factor-*β* (TGF*β*) signalling causes sensitivity to MEK1/2 inhibition. (**a**) pERK IHC on *VilCre*^*ER*^*Apc*^*fl/fl*^*Kras*^*G12D/+*^ (*Vil* *Apc*^*fl/fl*^*Kras*^*G12D/+*^) and *VilCre*^*ER*^*Apc*^*fl/fl*^*Kras*^*G12D/+*^*Tgfbr1*^*fl/fl*^ (*Vil* *Apc*^*fl/fl*^*Kras*^*G12D/+*^*Tgfbr1*^*fl/fl*^) intestine. Scale bars, 100 *μ*m. (**b**) Fluorescence lifetime changes for EKAREVnucl in *VilCre*^*ER*^*Apc*^*fl/fl*^ (*Vil* *Apc*^*fl/fl*^), *Vil* *Apc*^*fl/fl*^*Kras*^*G12D/+*^ and *Vil Apc*^*fl/fl*^*Kras*^*G12D/+*^*Tgfbr1*^*fl/f*^ CDS±EGF (50 ng/ml). (*Vil* *Apc*^*fl/fl*^ Δlifetime: −0.08±0.023, ****P<0.0001 by paired *t*-test, one-tailed; *Vil* *Apc*^*fl/fl*^*Kras*^*G12D/+*^: Δlifetime: −0.12±0.07, ****P<0.0001 by paired *t*-test, one-tailed; *Vil* *Apc*^*fl/fl*^*Kras*^*G12D/+*^*Tgfbr1*^*fl/fl*^ Δlifetime: −0.14±0.07, ****P<0.0001 by paired *t*-test, one-tailed). Data for each condition are mean of differences±S.D. for ⩾10 cells, *n*=1. *VilCre*^*ER*^*Apc*^*fl/fl*^*Kras*^*G12D/+*^+EGF *versus VilCre*^*ER*^*Apc*^*fl/fl*^*Kras*^*G12D/+*^*Tgfbr1*^*fl/f*^ +EGF, **P=0.003 by unpaired *t*-test, one-tailed. *VilCre*^*ER*^*Apc*^*fl/fl*^−EGF *versus VilCre*^*ER*^*Apc*^*fl/fl*^*Kras*^*G12D/+*^−EGF, *P=0.014 by unpaired *t*-test, one-tailed. *VilCre*^*ER*^*Apc*^*fl/fl*^*Kras*^*G12D/+*^−EGF *versus VilCre*^*ER*^*Apc*^*fl/fl*^*Kras*^*G12D/+*^*Tgfbr1*^*fl/f*^ –EGF, *P=0.049 by unpaired *t*-test, one-tailed. (**c**) Kaplan–Meier survival curve of *VilCre*^*ER*^*Apc*^*fl/+*^*Kras*^*G12D/+*^ treated with Selumetinib (*Vil* *Apc*^*fl/+*^*Kras*^*G12D/+*^, *n*=7) or Vehicle (*Vil* *Apc*^*fl/+*^*Kras*^*G12D/+*^, *n*=8), *P=0.023 by log-rank (Mantel–Cox) test and *VilCre*^*ER*^*Apc*^*fl+*^*Kras*^*G12D/+*^*Tgfbr1*^*fl/fl*^ mice treated with Selumetinib (*Vil* *Apc*^*fl/+*^*Kras*^*G12D/+*^*Tgfbr1*^*fl/fl*^, *n*=11) or Vehicle (*Vil* *Apc*^*fl/+*^*Kras*^*G12D/+*^*Tgfbr1*^*fl/fl*^, *n*=6). ****P<0.0001 by log-rank (Mantel–Cox) test. Treatment started 1day post induction with tamoxifen. (**d**) Quantification of top-down or bottom-up tumours in the small intestine (SI, left panel) and colon (right panel) of *VilCre*^*ER*^*Apc*^*fl/+*^*Kras*^*G12D/+*^ (*Vil* *Apc*^*fl/+*^*Kras*^*G12D/+*^) mice treated with Selumetinib or Vehicle. Error bars represent mean±S.E.M. (**e**) Quantification of top-down or bottom-up tumours in the SI (left panel) and colon (right panel) of *VilCre*^*ER*^*Apc*^*fl/+*^*Kras*^*G12D/+*^*Tgfbr1*^*fl/fl*^ *(Vil Apc*^*fl/+*^*Kras*^*G12D/+*^*Tgfbr1*^*fl/fl*^) mice treated with Selumetinib or Vehicle. Treatment started 1 day post induction with tamoxifen. Error bars represent mean±S.E.M., Mann–Whitney test, two-tailed

We next examined the mechanism by which *Tgfbr1* loss caused increased dedifferentiation and accelerated tumourigenesis. Previous studies have shown crosstalk between the MAPK and TGF*β* pathways.^27, 28^ pERK immunohistochemistry (IHC) showed a striking upregulation in the *VilCre*^*ER*^*Apc*^*fl/fl*^*Kras*^*G12D/+*^*Tgfbr1*^*fl/fl*^ mice compared with the *VilCre*^*ER*^*Apc*^*fl/fl*^*Kras*^*G12D/+*^ mice (Figure 6a). To measure precisely ERK activation, *VilCre*^*ER*^*Apc*^*fl/fl*^, *VilCre*^*ER*^*Apc*^*fl/fl*^*Kras*^*G12D/+*^ and *VilCre*^*ER*^*Apc*^*fl/fl*^*Kras*^*G12D/+*^*Tgfbr1*^*fl/fl*^ CDS were transfected with an ERK-FRET activity biosensor (we used the EKAREV biosensor consisting of an msECFP donor and YPet acceptor^29^) and treated with EGF for 45 min. A decreased lifetime indicates an activation of the nuclear biosensor. As expected, the fluorescence lifetime decreased following EGF administration in all three genotypes. Importantly, the activation of ERK was higher in the absence of *Tgfbr1* (Figure 6b).

To test functionally whether MAPK pathway activation was required for faster tumourigenesis, we treated *VilCre*^*ER*^*Apc*^*fl/+*^*Kras*^*G12D/+*^*Tgfbr1*^*fl/fl*^ mice with the potent allosteric MEK1/2 inhibitor Selumetinib (AZD-6244, ARRY-142886). Treatment started 1 day post induction and was able to reverse the acceleration of tumourigenesis conferred by *Tgfbr1* loss so that *VilCre*^*ER*^*Apc*^*fl/+*^*Kras*^*G12D/+*^*Tgfbr1*^*fl/fl*^ mice now had a median survival similar to vehicle-treated *VilCre*^*ER*^*Apc*^*fl/+*^*Kras*^*G12D/+*^ (Figure 6c). Interestingly, this effect was not restricted to the *VilCre*^*ER*^*Apc*^*fl/+*^*Kras*^*G12D/+*^*Tgfbr1*^*fl/fl*^ mice as tumourigenesis was also delayed in *VilCre*^*ER*^*Apc*^*fl/+*^*Kras*^*G12D/+*^ mice treated with AZD-6244.

To treat the *VilCre*^*ER*^*Apc*^*fl/+*^*Kras*^*G12D/+*^ mice for a similar timeframe as the *VilCre*^*ER*^*Apc*^*fl/+*^*Kras*^*G12D/+*^*Tgfbr1*^*fl/fl*^ mice, we started the treatment of the *VilCre*^*ER*^*Apc*^*fl/+*^*Kras*^*G12D/+*^ mice with Selumetinib 21 days post induction. Again, we were able to delay significantly tumourigenesis (Supplementary Figure 6a). Therefore, independent of the time of treatment (initiation or tumour outgrowth), Selumetinib could delay but not suppress tumourigenesis.

To assess if the reason for this is that Selumetinib affected the compartment from which tumours arose, we quantified top-down *versus* bottom-up lesions within the small intestine and colon of both genotypes (Figures 6d and e and Supplementary Figures 6b and c). MEK1/2 inhibition suppressed the formation of top-down lesions, suggesting that in both genotypes MAPK is important in the formation of this lesion type. However, mice still develop lesions from the crypt compartment, which led to the clinical signs. Thus, the MAPK pathway, following oncogenic *Kras* activation, is in part contributing to the increased plasticity of *Apc*-deficient cells.

## Discussion

The TGF*β* pathway is known to have both tumour-suppressing and -promoting effects in cancer. This makes it complex when trying to predict the outcome of therapies targeting the TGF*β* pathway. Here, we show a tumour-suppressive role of TGF*β* through its ability to limit tumour growth and dedifferentiation in the early stages of CRC. Recent studies have highlighted that plasticity of cells within the intestine act as stem cells and as potential tumour-initiating cells. Ours and other studies have highlighted that oncogenic *Kras* and inflammation can both drive plasticity in part via the NF-*κ*B pathway.^6, 8^ We were able to show that reducing NF-*κ*B directly or indirectly via *Rac1* loss could further limit transformation and reduce stem cell capacity.^6, 30^ An excellent study using a carbonic anhydrase Cre recombinase to target colonic enterocytes showed that *Apc* loss and *Kras* activation, but not *Apc* loss alone, can drive tumour growth in differentiated cells and generate ‘top-down’ tumours.^31^ Apart from these studies, very little is known about the pathways that confer plasticity in adult intestinal epithelium. Here, we show that loss of the TGF*β* signalling pathway induces the formation of more aggressive tumours, which are mainly responsible for the significant reduction of the overall survival. Interestingly, *Tgfbr1* loss also increases the ability of cells to dedifferentiate facilitating an increased number of top-down lesions.

It is important to note that, at least in our systems, *Kras* mutation is still necessary for dedifferentiation. Loss of TGF*β* signalling alone following *Apc* loss was not sufficient to drive dedifferentiation. Moreover, although *Tgfbr1* loss increases both the number of top-down lesions and presumably their speed of formation, top-down lesions still arise in *VilCre*^*ER*^*Apc*^*fl/+*^*Kras*^*G12D/+*^ mice that have functional TGF*β* signalling. We believe this might reflect a hierarchy of dedifferentiation capacity. Thus, dependent on the precise location where the lesions arise, they may be exposed to different levels of TGF*β* signals and this may be sufficient to block tumourigenesis from some cells, while others (potentially exposed to lower levels of TGF*β* signals) are able to form top-down tumours that once formed can grow regardless of these signals. It is interesting to note that despite high levels of p21 in many lesions that arise following *Apc* loss, deletion of *p21* does not modify *Apc* loss-induced tumourigenesis.^32^

It is interesting to note that other studies have suggested that activation of TGF*β* signalling might induce a stem cell phenotype^18^ and an EMT programme.^33, 34, 35^ However, our studies here indicate that exogenous TGF*β* reduces organoid-forming capacity *in vitro* in agreement with Alitalo laboratory’s work, where exogenous TGF*β* killed *Apc*-deficient crypts and reduced ISC markers *in vitro*.^36^

Previously, it has been shown that TGF*β* can positively regulate MAPK signalling.^37, 38^ In contrast, other studies fit with our model where the TGF*β* pathway can antagonize MAPK signalling. TGF*β* can regulate the expression of MKP2, which in turn attenuates ERK.^28^ Moreover, oncogenic *Kras*^*G12D*^ along with TGF*β* pathway inactivation has been associated with increased expression of epiregulin and ERBB1.^27^ Recently, it has been reported that *Tgfbr2* loss increases Ras/MAPK/ERK activation in primary keratinocytes leading to squamous cell carcinoma formation.^39^ Although our data support the model that TGF*β* signalling restrains pERK levels, the mechanism by which this is achieved requires further investigation.

The functional requirement for upregulated MAPK pathway following *Tgfbr1* loss was highlighted by MEK inhibition, which markedly reduced the number of tumours arising with top-down morphology. However, it is important to note that both *VilCre*^*ER*^*Apc*^*fl/+*^*Kras*^*G12D/+*^ and *VilCre*^*ER*^*Apc*^*fl/+*^*Kras*^*G12D/+*^*Tgfbr1*^*fl/fl*^ mice still developed tumours (adenomas in the case of *VilCre*^*ER*^*Apc*^*fl/+*^*Kras*^*G12D/+*^ mice and both adenoma/adenocarcinoma in *VilCre*^*ER*^*Apc*^*fl/+*^*Kras*^*G12D/+*^*Tgfbr1*^*fl/fl*^ mice) that have a bottom-up morphology presumably originating from the stem cell zone. This is an important finding showing that even in a simple mouse model system, where only *Apc* and *Kras* are mutated, *Kras* mutant tumours originating from the stem cell zone are resistant to MEK inhibition. These data are consistent with the lack of efficacy of MEK inhibitors as a single agent in advanced CRC carrying *KRAS* mutation (in both preclinical and clinical situations) as we would predict that inhibition of MEK would only work at a much earlier stage of tumourigenesis (e.g. during dedifferentiation).

**Figure 7:**
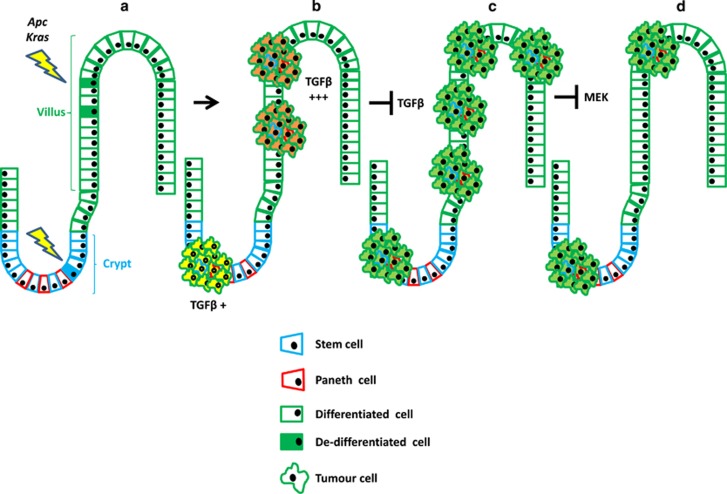
Schematic model of intestinal tumourigenesis. (**a**) Aberrant activation of WNT and KRAS signalling drives intestinal tumour formation both from crypts (bottom-up model) and villi (top-down model). (**b**) High levels of transforming growth factor-*β* (TGF*β*) signalling are present in the top-down model and this restrains tumourigenesis. (**c**) Loss of *Tgfbr1* promotes intestinal tumourigenesis. (**d**) Top-down tumours lacking of *Tgfbr1* are sensitive to MEK1/2 inhibition

In summary, we have defined that TGF*β* signalling limits dedifferentiation and tumourigenesis in cells carrying both *Apc* and *Kras* mutations (model in Figure 7). This is in part due to the gradients of TGF*β* signalling in the intestine. Higher levels of TGF*β* in the stroma further up the crypt–villus axis means that tumour-initiating cells in these regions are exposed to tumour-suppressive signals. Importantly, when tumours grow out from dedifferentiated cells, they seemed indistinguishable from tumours arising from the crypt. This suggests that once tumours have escaped the initial tumour-suppressive mechanism, the cell of origin does not affect the ongoing progression. Hence, mutational spectra might be a better way of predicting the initial cells of origin rather than transcriptomic data.

## Materials and methods

### Mouse experiments

All experiments were performed in accordance with UK Home Office regulations (licence 70/8646), which undergoes local ethical review at the Glasgow University (Glasgow, UK). *VillinCre*^*ER*^ experiments were performed on C57BL/6J mice and on mixed background (C57BL/6J/S129). A mix of males and females were used. Mice were induced at >20 g and between 6 and 12 weeks of age. The alleles used for this study were as follows: *VilCre*^*ER-T2*^,^40^ *Apc*^*580S*^,^41^ *Kras*^*G12D*^,^42^ *Tgfbr1*^*fl*^,^43^ *Smad4*^*fl*^,^44^ *Ctnnb*^*loxEx3/+*^^45^ and *Xbp1s*-*Cre*^ER-T2^.^6^ Recombination in the tumour model was induced using a single intraperitoneal injection of 80 mg/kg tamoxifen. Mice were aged until they showed clinical signs (anaemia, hunching and/or weight loss).

Recombination in the short-term model was induced using a single intraperitoneal injection of 80 mg/kg tamoxifen for 2 consecutive days. If *Kras*^*G12D*^ allele was present, recombination was induced using a single intraperitoneal injection of 80 mg/kg tamoxifen. *VilCre*^*ER*^*Ctnnb*^*loxEx3/+*^*Kras*^*G12D/+*^ and *Xbp1sCre*^*ER*^*Ctnnb*^*loxEx3/+*^*Kras*^*G12D/+*^ animals were induced with 1 mg tamoxifen for 5 consecutive days and sampled at day 7 (for *VilCre*^*ER*^) and day 21 (for *Xbp1sCre*^*ER*^). For the villi purification, mice were induced with intraperitoneal injection of 80 mg/kg tamoxifen for 2 consecutive days and sampled at day 1 post induction. The cultured villi treated with TGF*β*1 were isolated from mice that had been induced with a single intraperitoneal dose of 80 mg/kg tamoxifen.

In accordance with the 3Rs, the smallest sample size was chosen that could give a significant difference. Given the robust phenotype of the *Apc*^*fl/fl*^, and our prediction of the role of TGF*β* signalling, the minimum sample size assuming no overlap in control *versus* experimental is three animals. No randomization was used and the experimenter was blinded to drugs and genotypes.

### *In vivo* treatment

Selumetinib (AZD6244, ARRY-142886; AstraZeneca, Cambridge, UK) was administered in a concentration of 25 mg/kg two times daily by gavage in a vehicle of 0.5% hydroxymethylpropylcellulose and 0.1% Tween-80.

For proliferation analysis, mice were injected intraperitoneally with 250 *μ*l of BrdU (Amersham Biosciences/GE Heathcare, Buckinghamshire, UK) 2 h before being sampled.

### Crypt/villus purification, propagation and treatment

Small intestines were washed with PBS and opened longitudinally. Villi were removed with a glass coverslip, washed in PBS and centrifuged at 60 × *g* for 3 min to separate villi from single cells. A total of 50–70 villi, mixed with 20 *μ*l of Matrigel (BD Bioscience/Corning, Bedford, MA, USA), were plated in 24-well plates in Advanced DMEM/F12 supplemented with penicillin–streptomycin, 10 mM HEPES, 2 mM glutamine, N2, B27 (all from Gibco, Life Technologies, Paisley, UK), and EGF (50 ng/ml) and Noggin (100 ng/ml, both from Peprotech, London, UK). Crypts were isolated as described previously.^^46^^ Crypts mixed with 20 *μ*l of Matrigel were plated in 24-well plates and cultured in the medium described above.

Recombinant TGF*β*1 (Peprotech) was used at a final concentration of 5 ng/ml.

### Clonogenic assay

Organoid cultures were dissociated into single cells using TrypLE Express (Gibco, Life Technologies) for 30–45 min at 37 °C and 5000 cells were cultured in a 24-well plate in 20 *μ*l of Matrigel (BD Bioscience). The clonogenic potential was quantified after 72 h by microscope.

### Transplantation

For transplantation experiments, 50 spheroids were suspended in 100 *μ*l Matrigel (BD Bioscience) and injected subcutaneously into the flank of 6–8-week-old female athymic (CD-1) mice.

### Immunohistochemistry

IHC was performed on formalin-fixed intestinal sections. Standard IHC techniques were used throughout this study. Primary antibodies used for immunohistochemistry were as follows: pSMAD3 (1:50; 52903; Abcam, Cambridge, UK), BrdU (1:200; 347580; BD Biosciences), p21 (1/4; CNIO Madrid, Spain), pERK (1:100; 9101; Cell SignalingTechnology, Leiden, The Netherland) and CD44v6 (1:50; BMS145; Invitrogen/Thermo Fisher Scientific, Vienna, Austria). For each antibody, staining was performed on at least three mice of each genotype. Representative images are shown for each staining. BrdU scoring was performed in a blinded manner. The number of positive cells per half crypt was scored. For each analysis, at least 30 crypts were scored from at least three mice for each genotype. Representative images were selected for each scoring.

### RNAscope

*In situ hybridization* detection for *Tgfb1*, *Tgfbr1*, *Smad7*, *Lgr5* and *Olfm4* mRNA was performed using RNAscope 2.5 LS (Brown) Detection Kit (Advanced Cell Diagnostics, Hayward, CA, USA) on a Bond Rx autostainer (Leica, Milton Keynes, UK) strictly according to the manufacturer's instructions.

### Image analysis

For RNAscope and IHC analysis, intestinal slides were scanned on an SCN400F slide scanner (Leica, Milton Keynes, UK). The files were exported and analysed in Halo v2.0 Image Analysis Software (Indica Labs, Corrales, NM, USA). For pSMAD3 quantification, areas were manually defined around the more distinctly altered villus areas and the adjacent normal epithelium at day 3 post induction. For *Lgr5* and *Olfm4* quantification, areas were manually defined around the entire crypt and the more distinctly altered villus areas at day 3 post induction.

### RNA purification

RNA was isolated using a Qiagen RNeasy Mini Kit (Qiagen, Manchester, UK) according to the manufacturer’s instructions. DNA-Free Kit (Ambion/Thermo Fisher Scientific, Warrington, UK) was used to remove genomic DNA contamination, according to the manufacturer’s instructions.

### Microarray analysis

One microgram of total RNA isolated from intestinal tissue was reverse transcribed to cDNA and hybridized to Affymetrix Mouse Genome 430 2.0 Microarrays (Santa Clara, CA, USA). The data were preprocessed using affy package^47^ in R language, version 2.15.0. Background correction, quantile normalization and probe summarization were performed using the Robust Multiarray Analysis algorithm. Normalized data were analysed using the Limma package.^48^ Gene Ontology annotation was added using the GO.db package. Affymetrix Mose Gene ST 1.0 Chip (Santa Clara, CA, USA) has been used to analyse the *VilCre*^*ER*^*Ctnnb*^*loxEx3/+*^*Kras*^*G12D/*^+ and *Xbp1sCre*^*ER*^*Ctnnb*^*loxEx3/+*^*Kras*^*G12D/+*^ genotype. Data have been deposited in NCBI GEO (https://www.ncbi.nlm.nih.gov/geo/) and are accessible through GEO series accession numbers: GSE99077, GSE99100, GSE99408.

### Quantitative PCR (qRT-PCR)

One microgram of RNA was reverse transcribed to cDNA using a DyNAmo SYBR Green 2-Step qPCR Kit (Finnzymes, Espoo, Finland) in a reaction volume of 20 μl. The cycling conditions were as follows: 95 °C for 15 min, followed by 40 cycles of three steps consisting of denaturation at 94 °C for 15 s, primer annealing at the optimal temperature for 30 s and primer extension at 72 °C for 30 s. A melting-curve analysis was performed to demonstrate the specificity of each amplicon and to identify the formation of primer dimers*. β-Actin* and *Gapdh* primers were used to normalize for differences in RNA input.

### Transfection

For transfection experiments, intestinal spheroids were seeded in 96-well plates and transfected with EKAREV construct and Super PiggyBac Transposase expression vector (System Biosciences, Palo Alto, CA, USA) by using Magnetofectamine (OZ Biosciences, Marseille, France) according to the manufacturer's instructions. Subsequently, cells were incubated for 48 h at 37 °C and 5% CO_2_. After 48 h, organoids were selected using blasticidin.

### FLIM analysis

Fluorescence lifetime microscopy (FLIM) measurements were conducted on a spinning disk system, as described previously.^49^ Briefly, a Nikon Eclipse TE 2000-U microscope (Surrey, UK) with a Lambert Instruments LIFA attachment was used with a Yokogawa CSU 22 confocal scanner unit and a × 60 1.49 NA oil objective. FLIM-FRET for the msECFP donor was measured by frequency domain, with a 445 nm laser modulated at 40 mHz (100 mW, Deepstar; Omicron, Rodgau-Dudenhofen, Germany) was used together with a CFP filterblock (470/40) to detect CFP emission. Fluorescein (10 *μ*M in 0.1 M Tris-Cl, pH>10) was used as a reference standard with a known lifetime of 4.0 ns. The donor lifetime (msECFP^29^), *τ*, was analysed using the FLIM Software (version 1.2.12; Lambert Instruments, Groningen, Netherlands) drawing ROIs around the cell nuclei and recording the phase lifetime. Graph represents the lifetime variation (ns) before and after (45 min)±EGF (50 ng/ml) administration. In both cases, the mean of differences and standard deviation is calculated and represented *P*-values were calculated using Student’s *t*-test.

### Statistical analysis

Statistical analysis was performed with Minitab 17 Statistical Software (State College, PA, USA) or GraphPad Prism V6 Software (La Jolla, CA, USA) using one- or two-tailed Mann–Whitney tests and paired or unpaired Student’s *t*-tests (where appropriate).

### Data availability

The authors declare that all relevant data supporting the findings of this study are available within the article and its Supplementary Information files. Additional information can be obtained from the corresponding author (OJS).
