# Supplementary material for: TGFβ pathway limits dedifferentiation following WNT and MAPK pathway activation to suppress intestinal tumourigenesis
Source: Cell Death Differ. 2017 Jun 16;24(10):1681–93. doi: 10.1038/cdd.2017.92 (PMC5596428; doi:10.1038/cdd.2017.92)
Supplement: Supplementary Figure Legends [file cdd201792x1.docx]

**Supplementary Figure 1. TGFβ signaling is upregulated following WNT and oncogenic KRAS pathway activation. (a)** Significantly altered canonical pathways in VDS *versus* CDS. The transcripts were analysed by the ingenuity pathway analysis (IPA) software to identify the significantly perturbed growth proliferation pathways (x-axis). y-axis displays the -log of *P* value which is calculated by Fisher's exact test right-tailed. The yellow threshold line indicates the default significance, cut-off at *P*=0.05. The orange points connected by a thin line represent the Ratio. (**b)** Average nuclear optical density of pSMAD3 IHC in villus aberrant foci and adjacent normal epithelium analysed using Halo image analysis software. Error bars represent mean ± s.e.m., **P*=0.05 by Mann-Whitney test, one tailed, n=3 biological replicates. (**c)** *Smad7* RNAscope analysis showing increased positivity in *VilCre^ER^Apc^fl/fl^Kras^G12D/+^* (***Vil Apc^fl/fl^ Kras^G12D/+^***) compared to wild-type (***WT***) intestine. Scale bars, 100µm.

**Supplementary** **Figure 2.** ***Apc* deletion and** ***Kras^G12D/+^* activation drives dedifferentiation**. **(a)** Kaplan-Meier survival curve of a cohort of induced *VilCre^ER^Apc^fl/+^* (n=14) and *VilCre^ER^Apc^fl/+^Kras^G12D/+^* (n=12) mice. *****P*<0.0001 by Log-rank (Mantel-Cox) test. (**b)** H&E of small intestine (left panel) and colonic (right panel) top-down tumours from *VilCre^ER^Apc^fl/+^Kras^G12D/+^* (***Vil Apc^fl/+^ Kras^G12D/+^***) mice. Dashed lines highlight the top-down tumours. **(c)** H&E of small intestine (left panel) and colonic (right panel) bottom-up tumours from *VilCre^ER^Apc^fl/+^Kras^G12D/+^* (***Vil Apc^fl/+^ Kras^G12D/+^***) mice. **(d)** Schematic model of two possible routes for intestinal tumorigenesis. Either mutated stem or differentiated cells can start proliferating and produce either “bottom-up” or “top-down” tumours respectively. **(e)** pSMAD3 and **(f)** p21 IHC in small intestine and colonic top-down tumours from *VilCre^ER^Apc^fl/+^Kras^G12D/+^* (***Vil Apc^fl/+^ Kras^G12D/+^***) mice. **(g)** pSMAD3 IHC in bottom-up tumours from *VilCre^ER^Apc^fl/+^Kras^G12D/+^* (***Vil Apc^fl/+^ Kras^G12D/+^***) mice. Scale bars, 100µm.

**Supplementary Figure 3.** **Loss of *Apc* and *Tgfbr1* leads to invasive tumour formation**. **(a)** qRT-PCR analysis of *Tgfbr1* in villi purified from wild-type (***WT***) and *VilCre^ER^Apc^fl/fl^* (***Vil Apc^fl/fl^***) mice. Data are shown as ratios to the internal *Gapdh* control with error bars representing mean ± s.e.m., n=3 biological replicates. **(b)** RNAscope analysis of *Tgfb1* and **(c)** *Smad7* on crypts (left panel) and villi (right panel) from *VilCre^ER^Apc^fl/fl^* mice. Scale bars, 20 µm. **(d)** p21 IHC analysis on wild-type (***WT***) and *VilCre^ER^Apc^fl/fl^* (***Vil Apc^fl/fl^***) small intestine. **(e)** Morphology of villi freshly purified from *VilCre^ER^Apc^fl/fl^Tgfbr1^fl/fl^* mice at Day 0 (left panel) and Day 6 (right panel) in culture. Note that *VilCre^ER^Apc^fl/fl^Tgfbr1^fl/fl^* villi do not form spheroids, n=4 biological replicates. **(f)** Clonogenic capacity of single cells dissociated from *VilCre^ER^Apc^fl/fl^* and *VilCre^ER^Apc^fl/fl^Tgfbr1^fl/fl^* CDS. Error bars represent mean ± s.e.m., **P*=0.05 by Mann-Whitney test, one-tailed, n=3 biological replicates. **(g)** Kaplan-Meier survival curve of *VilCre^ER^Apc^fl/+^* (***Vil Apc^fl/+^*** n=14) and *VilCre^ER^Apc^fl/+^Tgfbr1^fl/fl^* (***Vil Apc^fl/+^ Tgfbr1^fl/fl^*** n=12) mice. Mice were culled at clinical endpoint. **P*=0.023 by Log-Rank (Mantel-Cox) test. **(h)** H&E of a representative invasive tumour from a *VilCre^ER^Apc^fl/+^Tgfbr1^fl/fl^* mouse. **(i)** E-cadherin IHC of an invasive tumour from a *VilCre^ER^Apc^fl/+^Tgfbr1^fl/fl^* mouse. TUM, Tumour. INV, Invasive front. Scale bars, 100µm.

**Supplementary Figure 4. Loss of *Tgfbr1* increases stem cell marker expression and invasiveness.** **(a)** qRT-PCR analysis of the stem cell markers *Lgr5* and **(b)** *Olfm4* in CDS purified from *VilCre^ER^Apc^fl/fl^Kras^G12D/+^* (***Vil Apc^fl/fl^ Kras^G12D/+^***) and *VilCre^ER^Apc^fl/fl^Kras^G12D/+^Tgfbr1^fl/fl^* (***Vil Apc^fl/fl^ Kras^G12D/+^ Tgfbr1^fl/fl^***) intestine. Data are shown as ratios to the internal *β-actin* control with error bars representing mean ± s.e.m., **P*=0.04 by Mann-Whitney test, one-tailed, n=3 biological replicates. **(c)** Clonogenic capacity of single cells dissociated from *VilCre^ER^Apc**^fl/fl^Kras^G12D/+^* and *VilCre^ER^Apc^fl/fl^Kras^G12D/+^Tgfbr1^fl/fl^* CDS. Error bars represent mean ± s.e.m., n=3 biological replicates. **(d)** *Lgr5* and **(e)** *Olfm4* RNAscope with relative positive area quantification in crypts and villi from *VilCre^ER^Apc^fl/fl^Kras^G12D/+^* (***Vil Apc^fl/fl^ Kras^G12D/+^***) and *VilCre^ER^Apc^fl/fl^Kras^G12D/+^Tgfbr1^fl/fl^* (***Vil Apc^fl/fl^ Kras^G12D/+^ Tgfbr1^fl/fl^***) intestine scored using Halo image analysis software. Error bars represent mean ± s.e.m., **P*=0.05 by Mann-Whitney test, one-tailed, n≥3 biological replicates. **(f)** H&E of a representative invasive tumour from a *VilCre^ER^Apc^fl/+^Kras^G12D/+^Tgfbr1^fl/fl^ (****Vil Apc^fl/+^ Kras^G12D/+^ Tgfbr1^fl/fl^****)* mouse. TUM, Tumour. INV, Invasive front. Scale bars, 100µm.

**Supplementary Figure 5. VDS lacking of *Tgfbr1* are tumorigenic. (a)** H&E **(b)** *Lgr5* RNAscope and **(c)** CD44v6 IHC analysis on an allografted tumour generated by the subcutaneous injection of 50 VDS purified from *VilCre^ER^Apc^fl/fl^Kras^G12D/+^Tgfbr1^fl/fl^* small intestine. Scale bars, 100µm.

**Supplementary Figure 6. *Apc* and *Kras* mutant tumours show a modest sensitivity to MEK inhibition. (a)** Kaplan-Meier survival curve of *VilCre^ER^Apc^fl/+^Kras^G12D/+^* mice treated with Selumetinib ***(Vil Apc^fl/+^ Kras^G12D/+^*** Selumetinib, n=8) or Vehicle (***Vil Apc^fl/+^ Kras^G12D/+^*** Vehicle, n=7). ***P*=0.0058 by Log-Rank (Mantel-Cox) test. Treatment started 21 days post-induction with tamoxifen. **(b)** Quantification of top-down or bottom-up tumours in the small intestine (**SI**) and **(c)** colon in *VilCre^ER^Apc^fl/+^Kras^G12D/+^* (***Vil Apc^fl/+^ Kras^G12D/+^***) mice treated with Selumetinib or Vehicle. Treatment started 21 days post-induction with tamoxifen. Error bars represent mean ± s.e.m., Mann-Whitney test, two-tailed.
