# Supplementary figures and images for: TGFβ pathway limits dedifferentiation following WNT and MAPK pathway activation to suppress intestinal tumourigenesis
Source: Cell Death Differ. 2017 Jun 16;24(10):1681–93. doi: 10.1038/cdd.2017.92 (PMC5596428; doi:10.1038/cdd.2017.92)

**Figure S1**

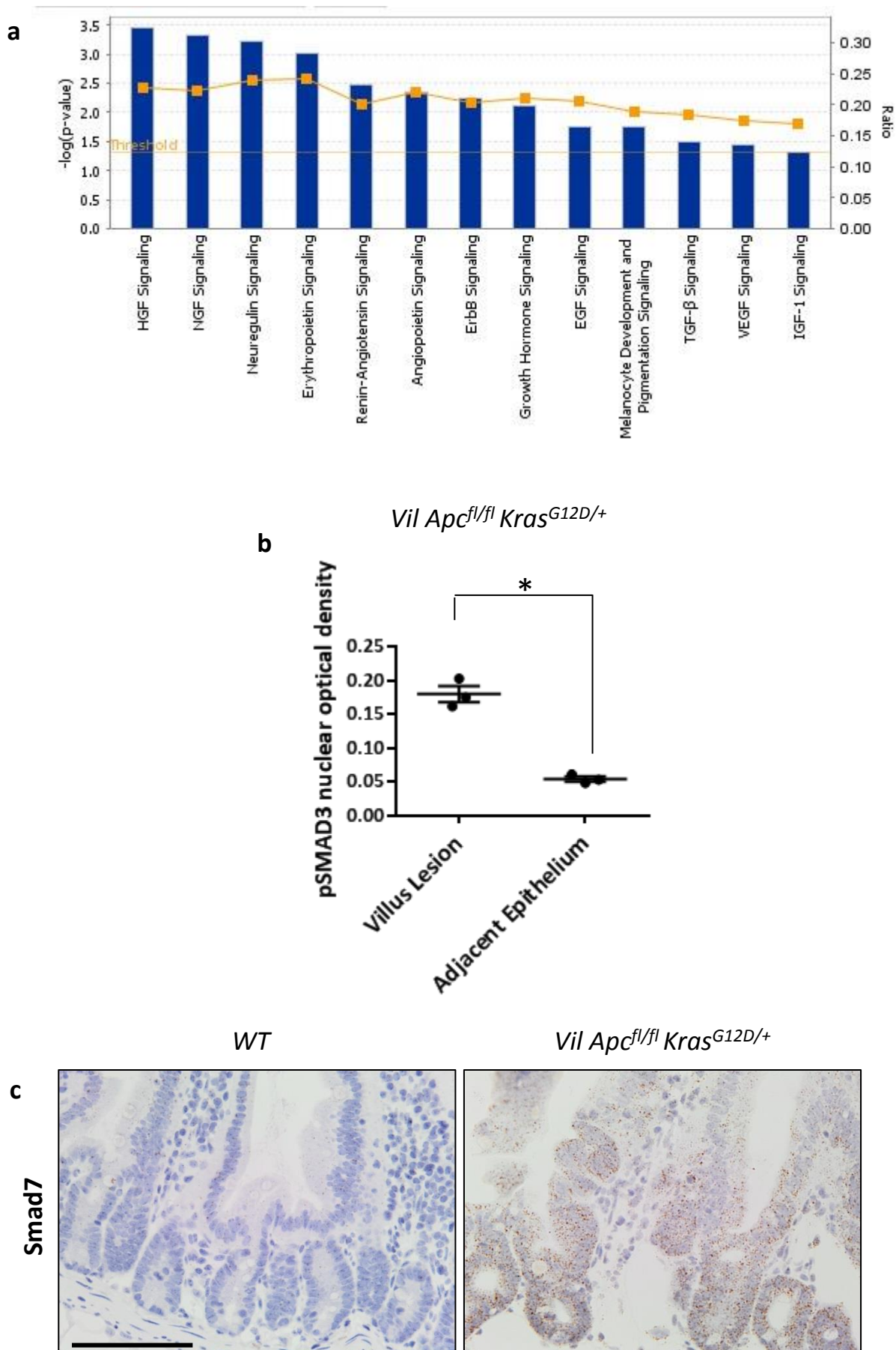

Supplement: Supplementary Figure S1 [file cdd201792x2.pdf]

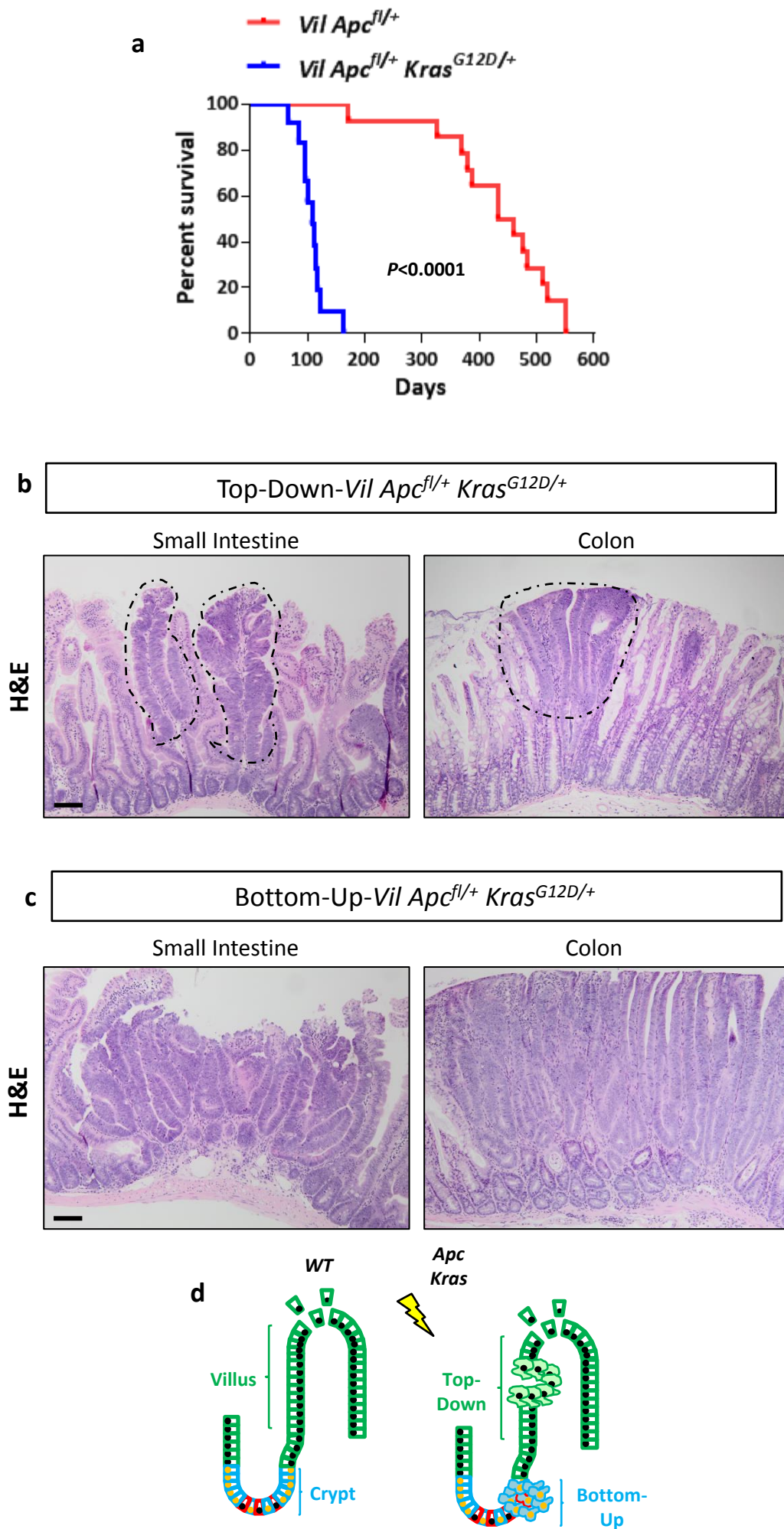

Figure S2

e

Top-Down-Vil *Apc<sup>fl/+</sup>* *Kras<sup>G12D/+</sup>*

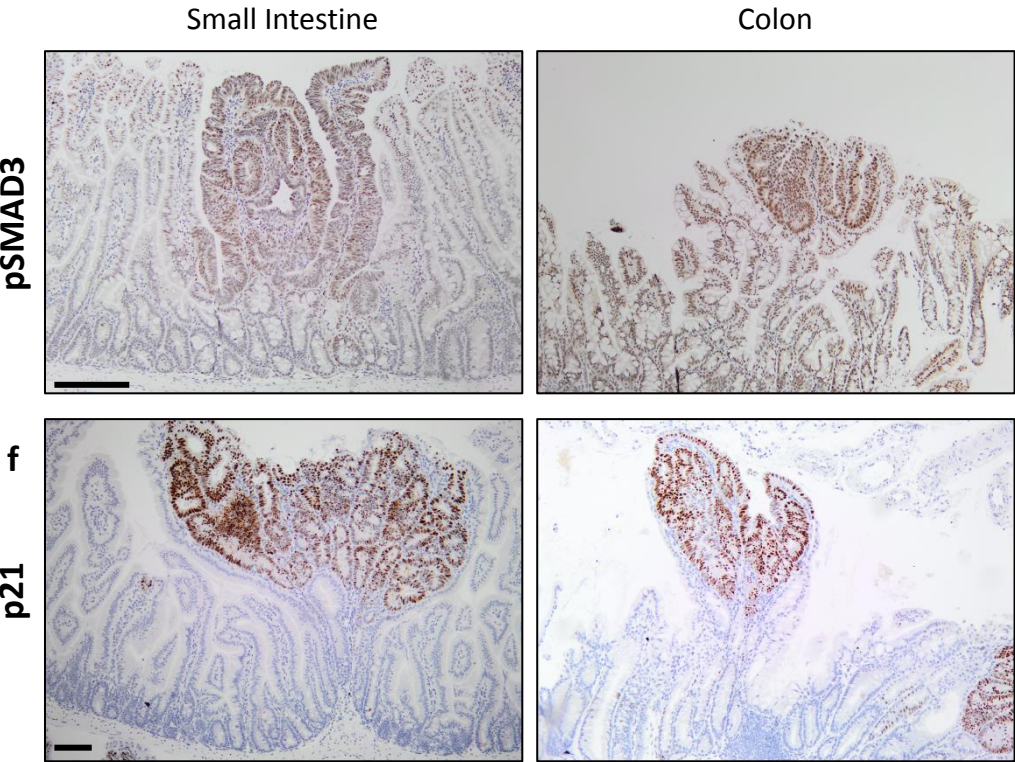

Bottom-Up-Vil *Apc<sup>fl/+</sup>* *Kras<sup>G12D/+</sup>*

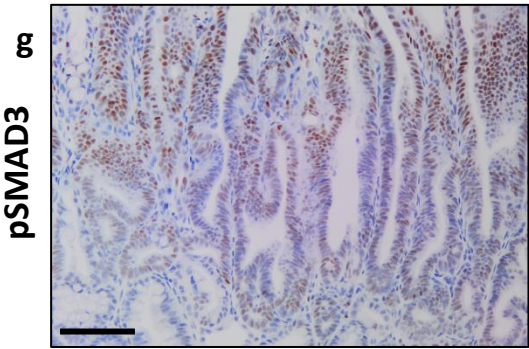

Supplement: Supplementary Figure S2 [file cdd201792x3.pdf]

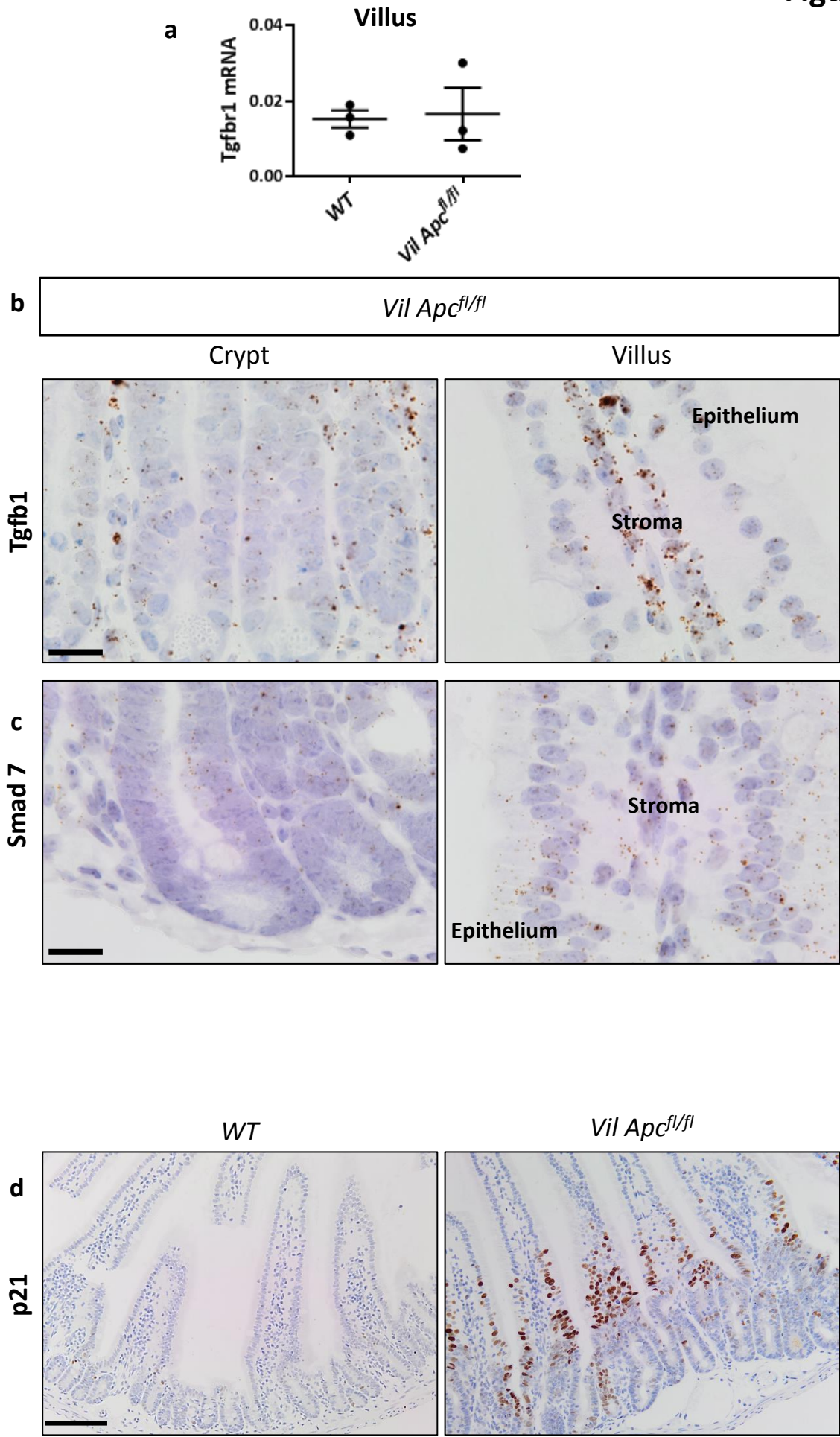

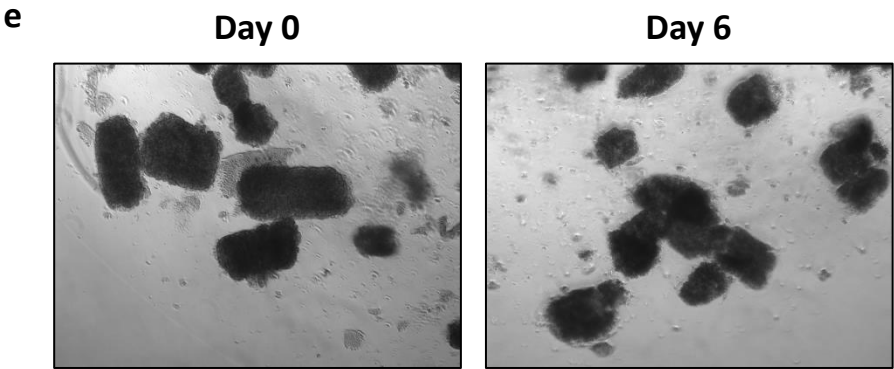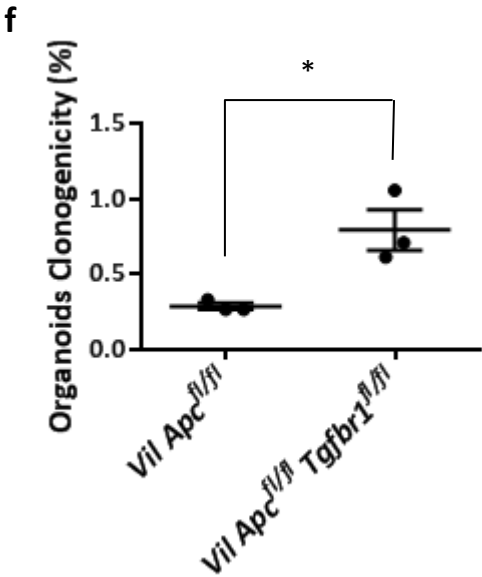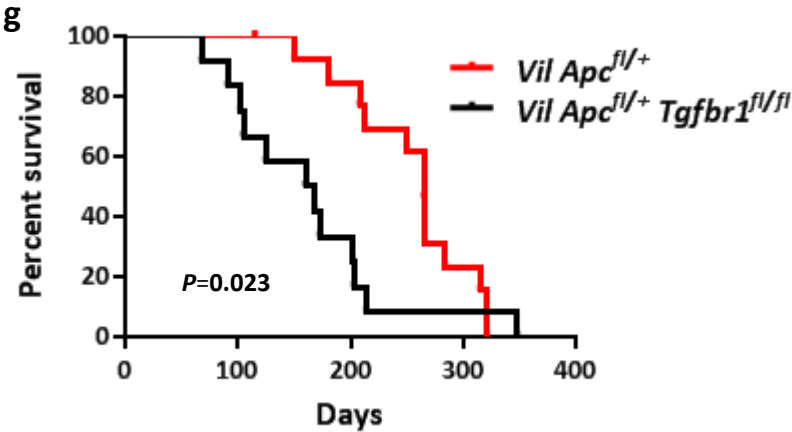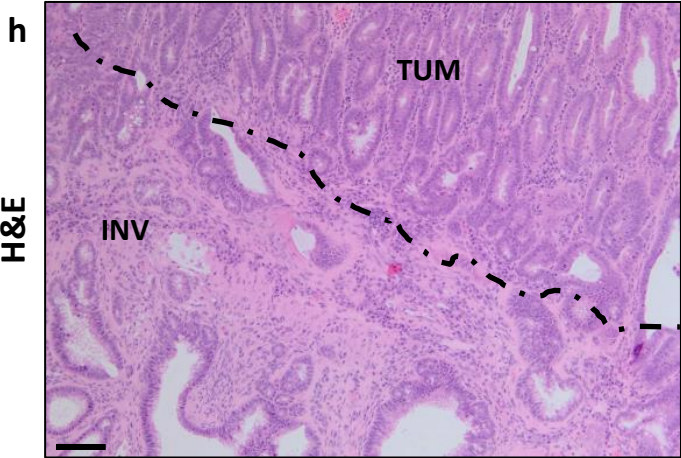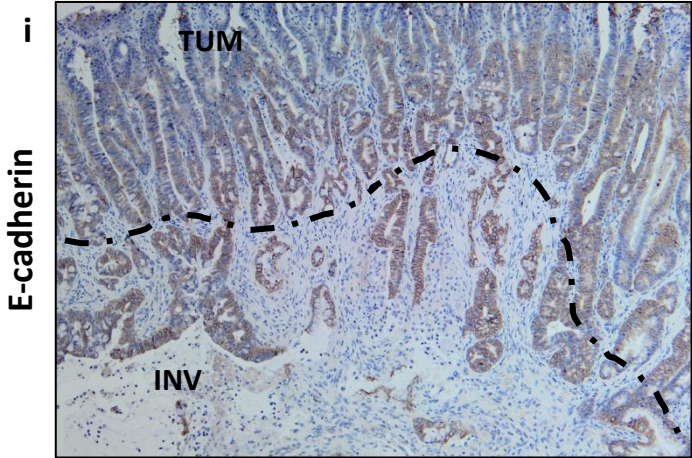

Supplement: Supplementary Figure S3 [file cdd201792x4.pdf]

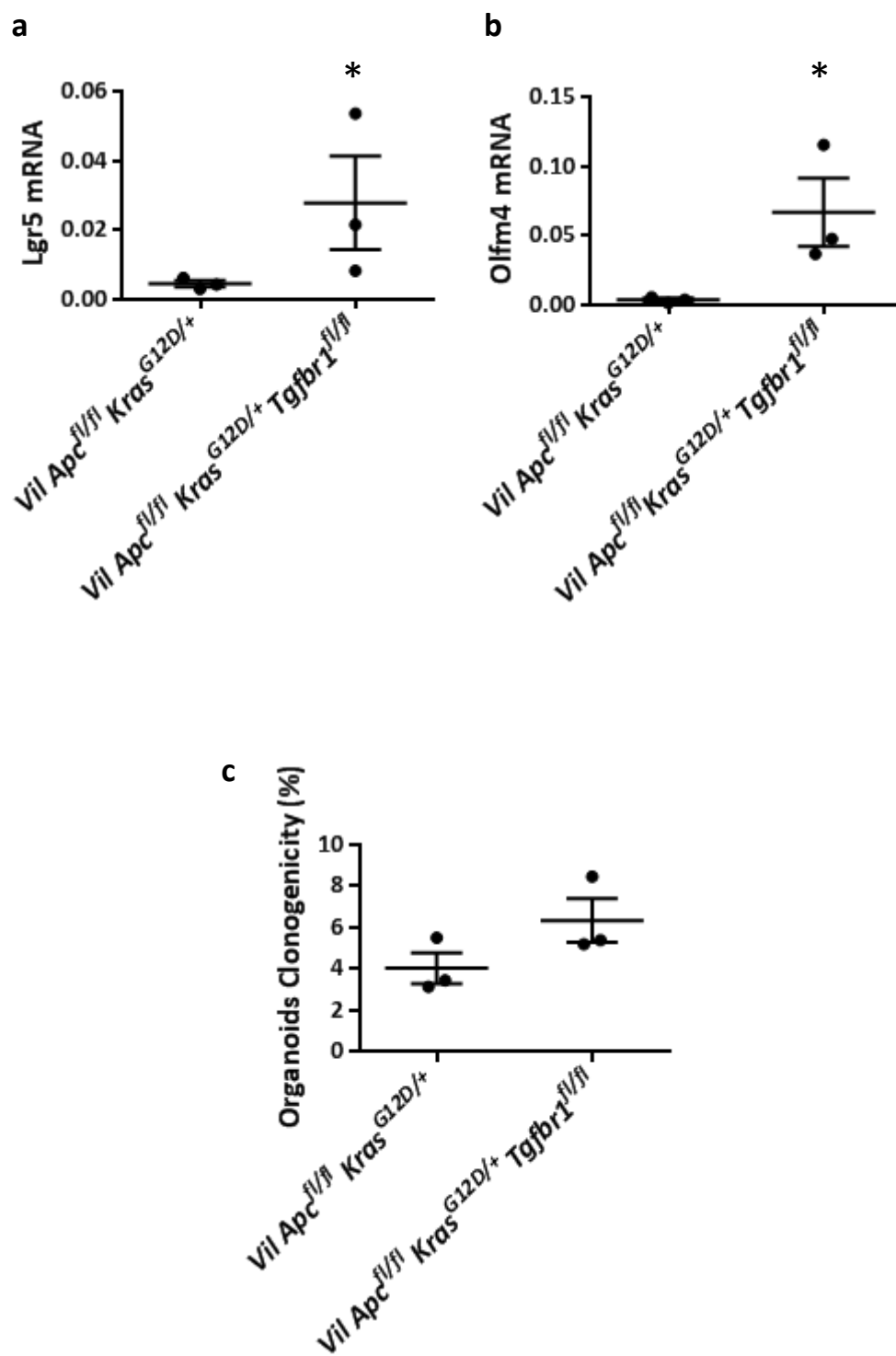

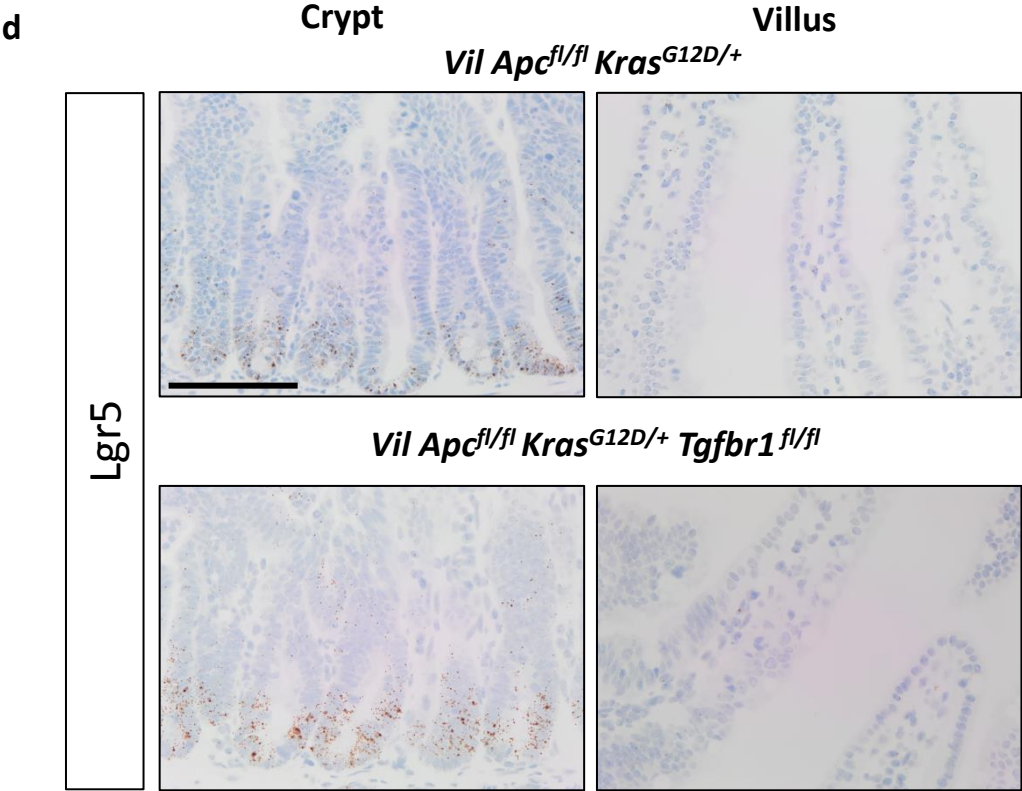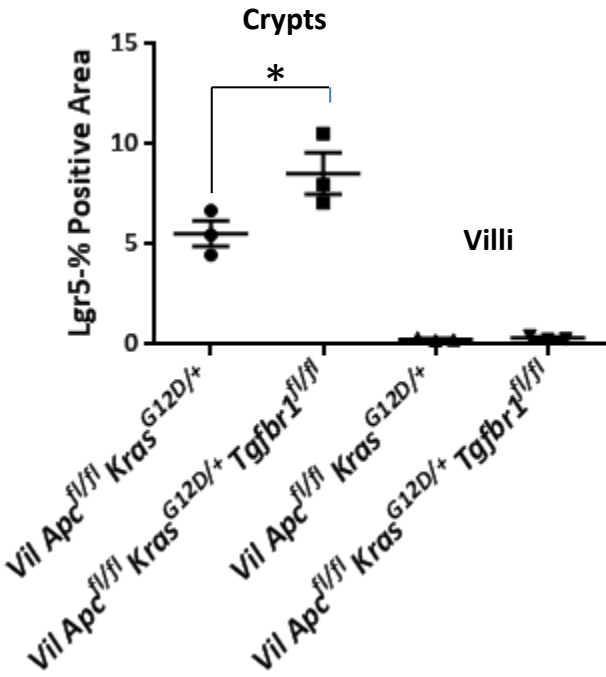

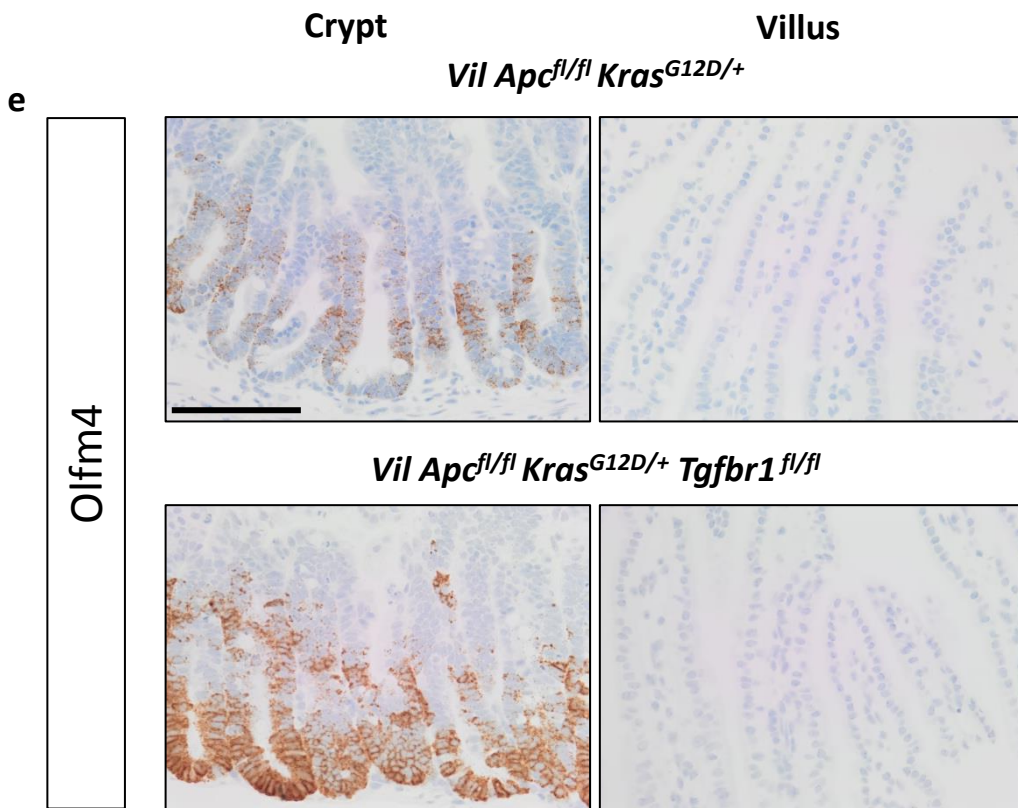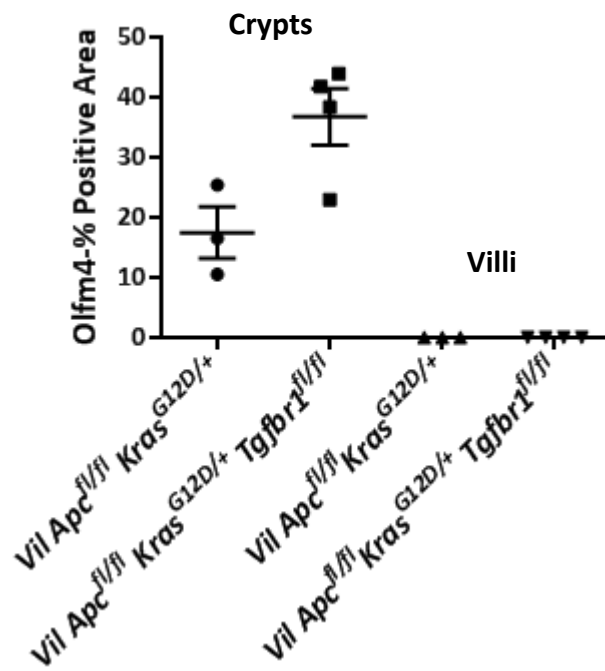

*Vil Apc<sup>fl/+</sup> Kras<sup>G12D/+</sup> Tgfbr1<sup>fl/fl</sup>*

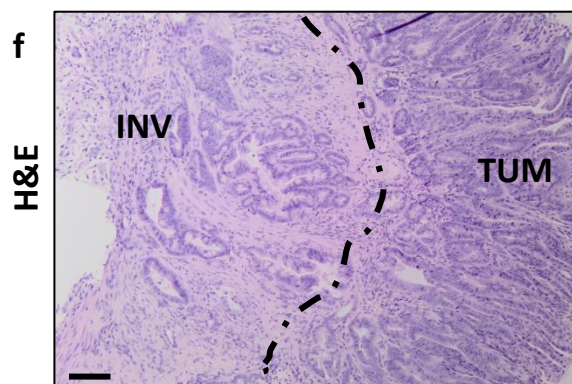

Supplement: Supplementary Figure S4 [file cdd201792x5.pdf]

*VDS-Vil Apc<sup>fl/fl</sup> Kras<sup>G12D/+</sup> Tgfbr1<sup>fl/fl</sup>*

**H&E**

**Lgr5**

**CD44v6**

**a**

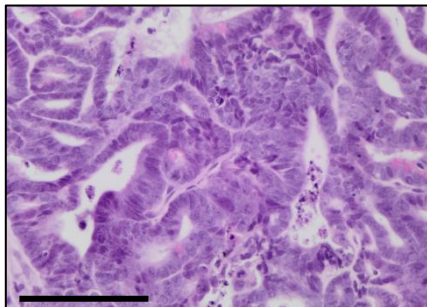

**b**

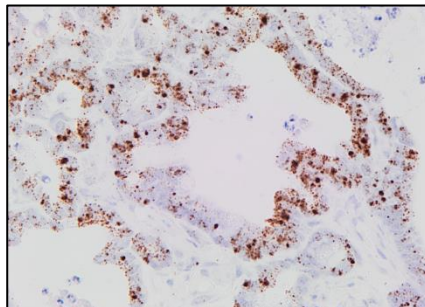

**c**

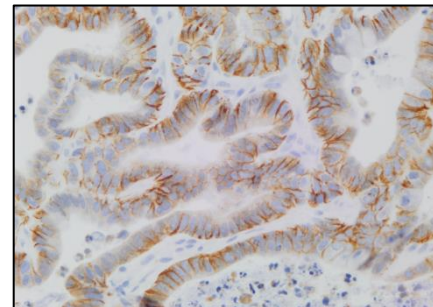

Supplement: Supplementary Figure S5 [file cdd201792x6.pdf]

Figure S6

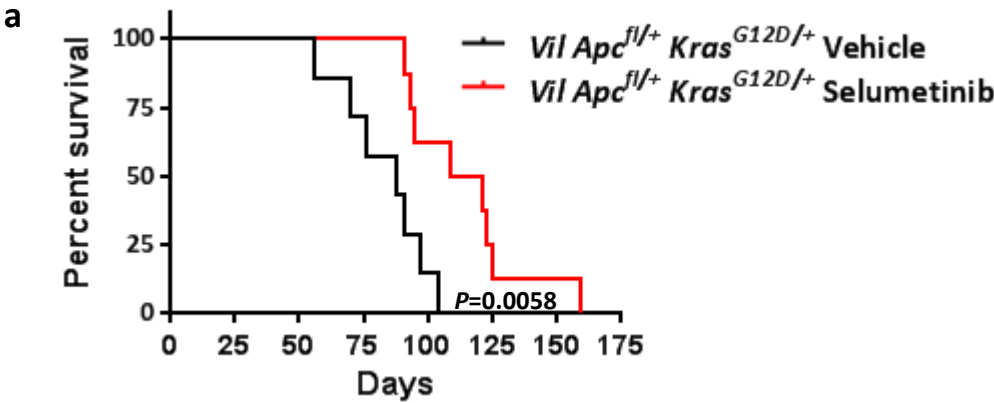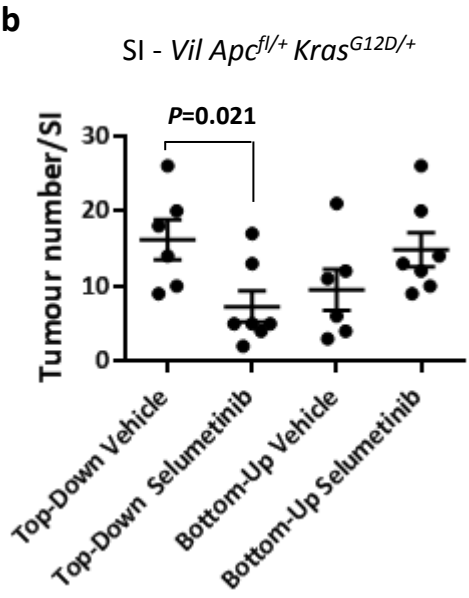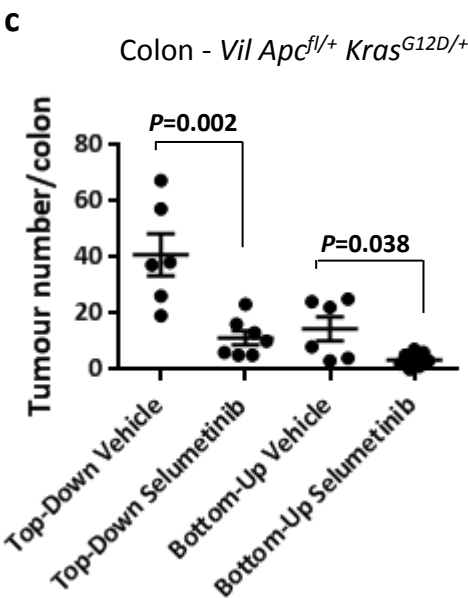

Supplement: Supplementary Figure S6 [file cdd201792x7.pdf]
